# Lifestyle-induced metabolic inflexibility and accelerated ageing syndrome: insulin resistance, friend or foe?

**DOI:** 10.1186/1743-7075-6-16

**Published:** 2009-04-16

**Authors:** Alistair VW Nunn, Jimmy D Bell, Geoffrey W Guy

**Affiliations:** 1 Metabolic and Molecular Imaging Group, MRC Clinical Sciences Centre, Hammersmith Hospital, Imperial College London, Du Cane Road, London, W12 OHS, UK; 2 GW pharmaceuticals, Porton Down, Dorset, UK

## Abstract

The metabolic syndrome may have its origins in thriftiness, insulin resistance and one of the most ancient of all signalling systems, redox. Thriftiness results from an evolutionarily-driven propensity to minimise energy expenditure. This has to be balanced with the need to resist the oxidative stress from cellular signalling and pathogen resistance, giving rise to something we call *'redox-thriftiness'*. This is based on the notion that mitochondria may be able to both amplify membrane-derived redox growth signals as well as negatively regulate them, resulting in an increased ATP/ROS ratio. We suggest that '*redox-thriftiness' *leads to insulin resistance, which has the effect of both protecting the individual cell from excessive growth/inflammatory stress, while ensuring energy is channelled to the brain, the immune system, and for storage. We also suggest that fine tuning of redox-thriftiness is achieved by hormetic (mild stress) signals that stimulate mitochondrial biogenesis and resistance to oxidative stress, which improves metabolic flexibility. However, in a non-hormetic environment with excessive calories, the protective nature of this system may lead to escalating insulin resistance and rising oxidative stress due to metabolic inflexibility and mitochondrial overload. Thus, the mitochondrially-associated resistance to oxidative stress (and metabolic flexibility) may determine insulin resistance. Genetically and environmentally determined mitochondrial function may define a '*tipping point*' where protective insulin resistance tips over to inflammatory insulin resistance. Many hormetic factors may induce mild mitochondrial stress and biogenesis, including exercise, fasting, temperature extremes, unsaturated fats, polyphenols, alcohol, and even metformin and statins. Without hormesis, a proposed *redox-thriftiness tipping point *might lead to a feed forward insulin resistance cycle in the presence of excess calories. We therefore suggest that as oxidative stress determines functional longevity, a rather more descriptive term for the metabolic syndrome is the *'lifestyle-induced metabolic inflexibility and accelerated ageing syndrome'*. Ultimately, thriftiness is good for us as long as we have hormetic stimuli; unfortunately, mankind is attempting to remove all hormetic (stressful) stimuli from his environment.

## Introduction

The nutritional milieu which modern humans have created for themselves is leading to rampant levels of obesity, type II diabetes (T2D) and insulin resistance [1]. This is resulting in a reduction in life expectancy.

The condition that precedes T2D, the 'metabolic syndrome', is currently defined as central obesity plus two factors: raised triglycerides (TGs), reduced HDL, hypertension and evidence of pathological insulin resistance, such as raised fasting plasma glucose (FPG, now defined as > 5.6 mM) or previous diabetes [2]. Sources of oxidative stress include fat overloaded cells in visceral adipose tissue (VAT) and the liver [3-6], and may represent 'metabolically triggered inflammation' or 'meta-inflammation' [7]. The metabolic syndrome is also associated with increased activity of the hypothalamic pituitary adrenal axis (HPA) and sympathetic nervous system (SNS), raised cortisol levels, and sex-specific alterations in androgens, which may represent an inability to adapt to an increased 'allostatic' workload [8]. The metabolic syndrome may therefore represent a metabolically inflexible phenotype, in which mitochondrial function and capacity for fuel usage are critical factors [9].

The metabolic syndrome is a continuum and may sit at the opposite end of the oxidative stress spectrum to the long-lived phenotype induced by calorie restriction [10]. A common feature of these two phenotypes is the involvement of the insulin/insulin-like growth factor axis, where the reduced activity associated with calorie restriction increases activity of the DAF 16/FOXO (forkhead) stress resistance transcription factors first described in *Caenorhabditis elegans *[11]; increased activity of these factors, in turn, can inhibit insulin signalling [12]. In evolutionary terms, insulin resistance may be good, as it ensures deposition of fat [13] and reduces oxidative redox signalling-induced stress, especially in muscle and adipocytes [14,15]. Indeed, thriftiness, which encapsulates insulin resistance, can be viewed as being genetically canalised and is a complex trait that most higher organisms exhibit. As well as an immediate response to famine, an emerging concept is that organisms can also be predisposed to it epigenetically via imprinting from their parents or even grandparents [16]. In human terms, different races, due to climate and geography, may well have slightly different predispositions to it – which may be reflected in differing fat distributions [17,18]. For instance, races with 'cold-genes' may be better protected [19]. An important organelle in this process is the mitochondrion: their ATP/ROS efficiency seems to improve during calorie restriction, but decreases in the metabolic syndrome and diabetes [20-22]. Mitochondria play a very important role in the aging process [23], and thus, modulation of oxidative stress.

We believe that it is now possible to provide a basic hypothesis to explain insulin resistance and the metabolic syndrome by studying redox signalling. In short, insulin resistance is determined by the ability to resist oxidative stress ('*redox-thriftiness*'), which is itself modulated by mitochondrial hormesis ('preconditioning') and thus, hormetic stimuli like physical activity and fasting. The development of the metabolic syndrome could then be defined by a "*thrifty-inflammatory tipping point*" – the point when insulin resistance goes from being thrifty (e.g. generally restricted to the musculature) to inflammatory (involving more tissues, such as adipose tissue). We propose that temporal and tissue specific insulin resistance is a friend as long as you live within your hormetic zone, but it may become your enemy in a modern sedentary environment. This paper outlines the underlying mechanisms relating to '*redox-thriftiness*', its relationship to an ancient redox signalling mechanism, and how it might be modified. The list of potential hormetic stimuli may extend to include plant polyphenols, unsaturated fats and alcohol, as well as some pharmaceuticals, such as metformin and the statins. Ultimately, the term 'metabolic syndrome' is not truly descriptive of the condition now afflicting a large fraction of mankind. We propose a more appropriate term might be the '**L**ifestyle-**I**nduced **M**etabolic **I**nflexibili**T**y and accelerated **AGE**ing', or, 'LIMIT-AGE' syndrome. The ultimate conclusion from this may be that 'thriftiness' is only bad for us without hormetic stimuli; a situation that very rarely occurred in prehistoric times – until humans made their environment almost totally risk and hormetic stress free. It is likely that any level of hormesis is better than none: this may be critical in reintroducing 'postive hormetic stressors' into a modern lifestyle.

## Insulin resistance and FOXO – built in safety?

Excessive insulin signalling can shorten lifespan by reducing a key stress resistance transcription factor, FOXO (forkhead box class-O 1). FOXO in turn can inhibit insulin signalling. Data might suggest that FOXO may well be very active in the metabolic syndrome as a protective response at the cellular level.

### Life is thrifty

Although much has been made of the 'thrifty genotype' [24], and its relationship to the metabolic syndrome [25], it is becoming clear that most animals, including humans, respond to prolonged fasting/starvation by improving feed efficiency, which is associated with selective tissue insulin resistance, hyperinsulinaemia on feeding, an accelerated rate of fat storage (i.e. catch-up fat), and probably, suppressed thermogenesis in certain organs/tissues [13]. This can result in a 'thrifty phenotype' – which can also be epigenetically imprinted to adapt future generations [26], resulting in thin-fat babies, who are more at risk in a modern environment [27,28]. More recently, an epigenetic/genetic canalisation hypothesis that amalgamates the thrifty genotype/phenotype hypotheses has been proposed. This hypothesis makes the point that life has always been exposed to feast and famine, so thriftiness is in fact an inherent property of many higher organisms and is resistant to mutational perturbations [16].

### Stress resistance inhibits insulin action and saves energy: the role of FOXO

Skeletal muscle insulin resistance in obese and type 2 diabetic patients is associated with increased activity of the stress c-jun N-terminal kinase (JNK) pathway [29]. Furthermore, transcriptional analysis of circulating white blood cells from type 2 diabetics shows that genes associated with JNK activity are upregulated, while those associated with oxidative phosphorylation are down-regulated [30]. Indeed, adipocyte-derived inflammation is thought to drive activation of JNK, which may well be one of the main underlying mechanisms of insulin resistance in the metabolic syndrome [31]. However, the JNK stress pathway is also associated with longevity because of the fact that it inhibits insulin signalling [32]. One of the ways it is thought to do this is by activating FOXO [33,34].

FOXO describes a family of transcription factors FOXO1, FOXO3a, FOXO4 and FOXO6, the mammalian orthologs of *C. elegans *DAF-16, which modulate the expression of genes involved in apoptosis, the cell cycle, DNA damage repair, oxidative stress, cell differentiation, as well as glucose metabolism. They undergo inhibitory phosphorylation by many protein kinases. Their activities are also modulated by acetylases, as well as deacetylases, such as the sirtuin, SIRT1(silent mating type information regulation 2 homolog 1), and by polyubiquitylation [35]. They are key in development, fasting, stress resistance and calorie restriction-induced longevity, whose function is suppressed by high insulin/IGF-1 activity [36]. FOXO activity can also increase glucose and lipids, decrease insulin, suppress growth and inflammation, and with AMPK, they increase appetite in response to fasting [37-39].

Increased expression/activity of FOXO can increase activity of PPAR γ co-activator 1 (PGC-1), which also plays a key role in longevity and the calorie restriction phenotype, in particular, it increases the expression of PPAR α [40]: 19% of the genes that are regulated during calorie restriction are modulated by PPAR α – including acute phase response (APR) genes [41]. PGC-1 is key in mitochondrial biogenesis and resistance to oxidative stress [42]. However, in muscle, exercise induced PGC-1 activation suppresses FOXO, but might result in a generalised anti-inflammatory effect induced by mitochondrial biogenesis [43]. FOXO is also important in autophagy, another important process in calorie restriction induced longevity [44].

Increased expression of FOXO in the liver, pancreas and adipose tissue has been shown to inhibit insulin signalling [12,45] and appears to induce a shift to fatty acid metabolism [46]. Importantly, they auto-amplify the insulin-Akt pathway by upregulating production of PI3k/Akt, so ensuring survival by stimulation of growth pathways in low nutrient conditions [33]. In white adipose tissue (WAT), FOXO1 appear to suppress the formation of new adipocytes, and in brown adipose tissue (BAT), suppress thermogenesis; expression of a mutant, inactive FOXO1 in the adipose tissue of mice seems to improve insulin sensitivity under high fat feeding and spare triglycerides, which is associated with increased thermogenesis and energy expenditure. In these mice there was a decrease in subcutaneous fat, but an increase in visceral fat – which was associated with an increased number of smaller adipocytes. There was also an increase in the number of adipocytes in BAT, which had increased expression of PGC-1 and uncoupling protein 1 (UCP -1) [47].

FOXO can inhibit leptin-induced appetite suppression in the hypothalamus [38] and insulin-induced beta cell proliferation in the pancreas [48]. The observation that insulin and leptin resistance go hand-in-hand, and in general are associated with obesity [49], does suggest that insulin and leptin can be viewed as anti-thrifty (they both increase energy usage and suppress appetite). Certainly, mice with reduced IRS-2 signalling are insulin resistance, hyperphagic and eventually develop obesity and T2D [50]. The fact that insulin and leptin signalling pathways cross-talk suggest a synergistic effect [51]. Hence, the finding that leptin resistance and increasing levels of leptin can also predict the metabolic syndrome [52], would suggest an evolutionary resistance paradigm to ensure continued energy seeking and storage behaviour – even when fat mass is increased. FOXO is very likely to play a key role in this.

### Redox negative feedback involving FOXO

ROS (and reactive nitrogen species, RNS) are not simply dangerous by-products, but essential components of cell signalling pathways [53,54]. Low levels of ROS seem to promote growth, whereas higher levels induce cell arrest [55]. ROS can active FOXO, which suggests that FOXO act as a negative regulator on increased ROS production [42,56-58]. FOXO are also modulated by AMPK – the archetypal energy sensor of the cell, which is itself activated by ROS [59,60]. FOXO activity is suppressed by insulin signalling in the short term, but this suppression is lost in the longer term – especially under stressful conditions, and involves a feed back loop that upregulates components of the Akt insulin signalling pathway [57]. Hence, excessive growth signalling it tightly modulated as it can result in excessive oxidative damage. Indeed, it has been proposed that feeding is associated with increased oxidative stress and can be viewed as inflammatory [61]. Glucose can also directly modulate FOXO function via O-linked-N-acetylglucosamine (O-GlcNAc), improving resistance to oxidative stress [62]. In *C. elegans*, overexpression of O-GlcNac transferase (OGT) can result in insulin resistance, whereas knocking out its function may improve insulin signalling and is associated with suppressed dauer formation and increased carbohydrate storage, but decreased lipid storage [63]. Indeed, increased flux through the hexosamine pathway has been known to be associated with insulin resistance (and thus, diabetes) for many years; addition of O-GlcNac is now a well described process to modulate the function of multiple proteins [64]. This would support the idea that FOXO can oppose insulin signalling and glucose-induced oxidative stress.

From an evolutionary perspective, some FOXOs are known to translocate to the nucleus in times of fasting and/or oxidative stress, so improving somatic protection, but reducing energy allocation to growth and reproduction. However, after extended fasting, there is evidence, at least in *C. elegans*, that they translocate back out of the nucleus in what appears to be an Akt-Pi3K dependent mechanism. The explanation for this appears to be that somatic protection comes at an energy cost (e.g. manufacture of anti-oxidant proteins), and once anti-oxidant defences have been improved, the process is downregulated [65]. Thus, continual growth signalling and excessive calories might cause FOXO to remain active and thus continue to be active in the metabolic syndrome.

### FOXO and nature of thriftiness

Failure to eat is a strong negative selective pressure, which has likely led to an imbalance between orexigenic (stronger) and anorexic (weaker) signals, leading to high feed-efficiency and a propensity to store fat [66-68]. As both inflammation, and feeding (via increased Akt signalling), might act to suppress FOXO activity, but FOXO activity may be important in resistance to stress via suppression of ROS – it could be argued that FOXO must be a powerful counter-regulatory mechanism. Certainly, TNF-α is known to activate FOXO, which can then induce apoptosis [69]. However, inhibitor of kappa B kinase (IκBK), which also activates nuclear factor kappa B (NF-κB), can also inhibit members of the FOXO family [70], implying a finely tuned response around modulation of potentially energy consuming immune responses. It is therefore of interest that a high fat diet can induce a pro-inflammatory response in the hypothalamus and insulin resistance [71], while chronically elevated levels of leptin can also induce leptin resistance – which may be part of an obesity-driven vicious cycle [72]. These observations could be partly explained by FOXO activity.

Two recent pieces of research suggest that redox is integral to the appetite/anorexic mechanism, and integrate this action with the endocannabinoid system (ECS). Via activation of AMPK, ghrelin results in increased mitochondrial oxidation of fatty acids, increased ROS and a concomitant increase in anti-ROS mechanisms, including transcription of UCP-2 and increased mitochondrial biogenesis. This has the overall effect of reducing mitochondrial membrane potential and ROS production. Importantly, it appears that orexigenic neuropeptide Y/agouti-related protein (NPY & AgRP) neurons become active in a low ROS situation, which is the opposite of anorexigenic pro-opiomelanocortin/cocaine- and amphetamine-regulated transcript (POMC) cells, which appear to rely more on glucose and are more active at higher ROS levels. Hence, the orexigenic circuit may rely more on fatty acids, whereas the anorexic one relies more on carbohydrate [73]. In another study, via activation of PKC, ghrelin was found to activate diacylglycerol lipase (DGL), which increases 2-arachidonoylglycerol (2-AG), so activating the CB-1 receptor: this then auto-activates itself in a positive feed-forward loop involving PKC again. Without the involvement of CB-1, ghrelin becomes ineffective [74].

This data suggests that the ECS is involved in altering cellular redox and that this may link in with FOXO and mitochondrial function, both of which are involved in appetite control. Furthermore, it also suggests that orexigenic circuits may well rely on lower levels of redox to function, whereas anorexic ones rely on higher levels. Hence, excessive calorie intake, especially of high glycaemic index carbohydrate, might induce the anorexic circuit to fail or down regulate to protect itself, leaving the orexigenic one intact, as it has better oxidative stress resistance; it would also be more likely to function during starvation, when lipids become the predominant fuel in the body. It would also support the use of low carbohydrate diets, which can often reverse many symptoms of the metabolic syndrome [75].

In summary, the above support the hypothesis that excessive insulin (and leptin) signalling can increase oxidative stress. Hence, resisting the signalling is a vital counterbalance in survival and fulfils a basic evolutionary paradigm of coupling food seeking and storage behaviour with resistance to oxidative stress. Thus, FOXO may well epitomise thriftiness, and the default setting to continual stress (e.g. over-eating) must be to maintain its activity.

## Mitochondria, hormesis and the metabolic syndrome: '*redox-thriftiness*'

A notable finding in the metabolic syndrome and T2D is that muscle mitochondrial function seems to be reduced [22]. This mitochondrial dysfunction is also found in other tissues, including the vascular endothelium and may be related to mitochondrial overload by excessive glucose flux through the electron transport chain (ETC) [76]. Adipocytes can also suffer from fatty acid overload, leading to mitochondrial dysfunction and oxidative stress. Under normal circumstances, adipocytes may be able to burn off excessive fat as heat, so preventing lipotoxic damage to other organs [77].

As insulin signalling plays a vital role in controlling mitochondrial function, this suggests that insulin resistance, reduced mitochondrial function, and the metabolic syndrome are all linked. As mitochondria are potentially wasteful of energy, it is likely that reduced food availability would, via natural selection, select for the minimal functional mitochondrial density needed to produce energy – 'symmorphosis' or economy of design [78]. In contrast, it is now becoming apparent that various stressors, such as exercise, fasting, and some polyphenols, can induce mitochondrial biogenesis via a process called 'mitohormesis' – all of which are associated with improved functional longevity [79]. The most important of these may well be exercise, and it has been suggested that the increased inflammatory tone seen in the metabolic syndrome may be due to reduced PGC-1 activity, which has a strong anti-inflammatory effect [43].

The ecological stress theory of ageing suggests that optimal survival (longevity) is probably reliant on a degree of stress to stimulate resistance to these stressors; in essence, a (mild) degree of stress stimulates the cell (organism) to improve its anti-stress mechanisms, which by and large result in an improved ability to resist oxidative stress and upregulate DNA repair – this process is known as 'hormesis'. These stressors include heat, cold, calorie restriction, excessive gravity, exercise and irradiation. As these stimuli result in long lasting effects, they might be expected to slow the ageing process. The downside may therefore be that removal of these stresses might be expected to reduce biological fitness; in their optimal environment, animals normally live in a 'hormetic zone' [80] – which could also be described as the 'Goldilocks' zone, neither too comfortable, but not too harsh. In this light, mitohormetic stimuli must be critical for optimal functioning. In order to shed light on the nature of insulin resistance, we have developed the concept of mitochondrially-driven '*redox-thriftiness*'. Underlying this is an emerging concept that the mitochondrium plays a critical role in the modulation of redox signalling, and thus, insulin resistance. Therefore, by improving mitochondrial function (defined by the ATP/ROS ratio), not only is metabolic flexibility improved, but inflammation and insulin resistance can be reduced, as the signalling pathway has less negative impact on intracellular redox.

### Mitochondrial amplification of membrane-derived redox signals

Many membrane-based receptors and kinase-based pathways (e.g. p38 MAPK [mitogen activated protein kinase], JNK and IKK/NF-κβ) may signal via or be modulated through redox-based mechanisms [81,82]. MAPKs are a large family of kinases that control cellular proliferation and arrest in a redox-dependent manner: low levels of hydrogen peroxide result in proliferation, whereas increased levels suppress growth and eventually, induce apoptosis. Thus, mitochondrial production of hydrogen peroxide is critical in controlling cell growth and arrest. However, it now appears that MAPKs are also located in the mitochondrium, and that their translocation to the nucleus, or cytosol, or even back into the mitochondrium, is dependent on oxidation status. Thus, different levels of oxidation result in different patterns of MAPK redistribution throughout the cell. As mitochondrial dysfunction is common in cancer cells, this might suggest that the inability to increase peroxide production would maintain cell growth [83]. Mitochondria can also amplify ROS signals, for instance, ROS can inhibit the mitochondrial permeability transition pore (MPTP), resulting in increased mitochondrial ROS, which can be propagated throughout the cell [84]. Moreover, mitochondria are also critical in calcium signalling: calcium can activate mitochondrial function, but calcium plus other physiological stimuli can also increase ROS release – a 'two-hit' mechanism that might escalate normal physiology to pathology [85].

ROS is not the only redox signal: reactive nitrogen species (RNS), as well as hydrogen peroxide and carbon monoxide, are also important. These superoxide radicals may have slightly different functions. For instance, membrane-derived nitric oxide (NO) is a potent stimulator of mitochondrial biogenesis and may work by inhibiting mitochondrial function as a competitor for oxygen at cytochrome oxidase; this may also induce production of mitochondrial nitric oxide – suggesting an amplification effect. It can therefore modulate energy production [54]. Indeed, it has been suggested that it can fine tune the bioenergetics of the cell, inducing a mild 'metabolic hypoxia' that induces cytoprotection [53]. Carbon monoxide, produced by haem oxygenase, may also play a similar role by inhibiting cytochrome oxidase and increasing ROS, resulting in mitochondrial biogenesis [86].

One of the concepts that emerges from the above is that low level redox signalling is important in maintaining critical cellular function, while a slight increase induces cytoprotection – but too much will induce cell death. Certainly, the cell cycle is now thought to be controlled by redox [87]. An example of this may come from the role of inducible nitrogen oxide synthase (iNOS) versus endothelial NOS (eNOS): iNOS is very important in pathogen resistance, as it can induce large amounts of NO. When combined with ROS, it becomes highly toxic in the form of peroxynitrite [88]. TNFα can inhibit eNOS function in adipose and muscle tissue [89], but can increase iNOS. It has now been proposed that a 'yin-yang' eNOS/iNOS balance plays an important role in modulating insulin resistance. Insulin-stimulated production of NO by eNOS in the vasculature ensures capillary bed dilatation in muscles, so enabling efficient glucose dispersal, however, this process stops working when there is either too little eNOS activity (e.g. during insulin resistance, or due to eNOS polymorphisms), or too much iNOS activity (inflammation), corresponding to too little, or too much NO, respectively [90]. Thus, both ROS and RNS (NO) can not only be amplified by the mitochondrium, but they also play a vital role in insulin sensitivity or resistance, depending on their concentration. High levels of oxidative stress are well known to be associated with inflammation and insulin resistance, but importantly, oxidative stress can also be an important stimulus for mitochondrial biogenesis – which can thus be viewed as a negative feedback mechanism, and is discussed in the next section.

### Mitochondria, free radicals, and calorie restriction

Calorie restriction induces eNOS, which may be an important inducer of the mitochondrial biogenesis observed in calorie restriction involving PGC-1 α [20,21,91]. One explanation for this is an increase in autophagy, which recycles damaged components and results in newer, more efficient organelles. This process is modulated, in part, by mTOR and FOXO [44,92]. The resulting mitochondria have a reduced membrane potential (deltapsi), produce less ROS, use less oxygen and exhibit an improved ATP/ROS ratio – which might explain the decrease in energy expenditure induced by calorie restriction [21]. PGC-1α function is also modulated by AMPK [93], calcium [94], mTOR [95], FOXO [40], and the sirtuins [96].

The sirtuins are NAD-dependent deacetylases that are upregulated during calorie restriction, and appear to be important in stress resistance and longevity. There are several members, some of which locate to the mitochondrium. One of the reasons they are becoming the focus of much research is that many plant polyphenols, such as resveratrol, can mimic calorie restriction-induced longevity – possibly by modulating sirtuin function/expression; at least two downstream targets are p53 and FOXO [97,98]. It is now clear that many of these polyphenols can induce mitochondrial biogenesis, which may be associated with direct activation of sirtuins, or indirectly via their increased expression [99].

The evolutionary strategy for increased mitochondrial mass and/or efficiency during calorie restriction may revolve around an enhanced ability to utilise fatty acid oxidation, which in muscle, maintains the ability to move and maintain body temperature. Interestingly, fatty acid oxidation is less reliant on complex 1 of the ETC (the main source of ROS). This may result in a slightly reduced ATP output – which may be another reason for increasing mitochondrial density [100]. The overall effect of calorie restriction is to enhance the organism's chance of survival by reducing oxidative stress and ROS, while switching to easily stored fatty acids. This would support the hypothesis that FOXO shifts metabolism towards burning fats.

Recent data suggest that glucose restriction of *C. elegans *increases its lifespan via an induction of respiration, which is associated with an increase in mitochondrial ROS and activation of AMPK; the inference is that glycolysis, although inefficient, produces no ROS – so reducing glucose leads to a hormetic stimulus [101]. Hence, it is likely that reducing available carbohydrate (starvation), induces a switch to mitochondrial respiration and increased ROS, which in turn, activates mitochondrial biogenesis. This fits well with the observation that calorie restriction/starvation can induce insulin resistance, which is associated with an increase in IMTG – so ensuring a switch to fatty acids as fuel. As suggested by the *C. elegans *data, it is now thought that AMPK is critical in the mitochondrial bioenergetic process, especially during exercise, as it can activate PGC-1α [102]. This would support data that it can improve the ability to oxidise fatty acids and be able to offset fatty acid-induced insulin resistance, such as in muscle [103]. Conversely, excessive glucose can inhibit its function and thus, induce insulin resistance, in muscle and liver [104]. AMPK is also important in stimulating fatty acid oxidation in adipose tissues, and is activated by exercise and hormones, such as leptin and adiponectin [105]. Critically, inflammatory cytokines, such as TNFα, are thought to inhibit its function [106]. AMPK may also modulate the function of the FOXO transcriptional factors, implying coordination of resistance to oxidative stress and energy metabolism [107]. There is thus a clear correlation between improved mitochondrial function and calorie restriction: given that PGC-1α also upregulates anti-oxidant capacity, then increasing mitochondrial density is probably likely to suppress redox growth signalling. In calorie restriction and/or stress, two critical nutrient sensors, SIRT1 and AMPK, may well act concordantly to do this [108]. As indicated, one of the strongest stimulators of PGC-1 is exercise, hence, a lack of exercise may well result in rising inflammatory tone [43].

### Insulin control of mitochondrial function

The above suggests that insulin must have an effect on mitochondrial function – perhaps by inducing oxidative stress. Insulin signalling utilises hydrogen peroxide, which is at least partly generated by the mitochondrial respiratory chain and is important in the autophosphorylation of the insulin receptor [109]. Additionally, mTOR, which is part of a well conserved serine/threonine kinase pathway that regulates cell growth in response to nutrient status, also modulates mitochondrial function. It has a pro-survival and proliferative function. Inhibition of this pathway using rapamycin lowers mitochondrial membrane potential, oxygen consumption, and ATP synthetic capacity [110]. However, mild inhibition of the mTOR pathway may also be associated with increased longevity; it appears to be downregulated in times of stress by factors such as p53 or AMPK [111]. Its effects on mitochondrial function are thought to work through a complex with PGC-1α and another transcription factor, ying-yang 1 [95]. The mTOR pathway can also self inhibit via the s6 kinase [112]. All together, it is likely that insulin can promote mitochondrial biogenesis as part of a general proliferative function, while stressors promote it as a mechanism to ensure increased resistance to stress. Certainly, glucocorticoids can upregulate PGC-1α in muscle, and can directly modulate mitochondrial function, including mitochondrial biogenesis – which may involve glucocorticoid receptors in the mitochondrion itself [113-115]. Critically, it appears that inflammation can both suppress insulin signalling and damage mitochondria (via TNF-α), but this in itself might be a potent mitochondrial biogenic signal: LPS treatment of cardiomyocytes depresses mitochondrial function, but results in activation of PGC-1α [116].

### *Redox-thriftiness*: 'mitoamplification'

The key to '*redox-thriftiness' *is that mitochondria can both amplify, and suppress, redox signalling. For instance, it is possible that a small number of high potential mitochondria may amplify the redox growth signal more strongly than a larger number of low potential mitochondria. In light of this, we propose the concept of '*redox-thriftiness*' to explain the molecular basis for insulin resistance. Due to the need to both resist oxidative stress, and save energy, a rapid mitochondrial amplification of redox growth signals would result in rapid negative regulation of the signal. This phenotype would ensure supply of energy to the brain, energy storage (channel lipids to adipose), a heightened inflammatory response, but reduce insulin signalling (as it is potentially life-shortening and may result in excessive mitochondrial biogenesis). However, with hormetic stimuli, mitochondrial function (and probably mass) would improve (increase), so increasing resistance to oxidative stress and would improve insulin sensitivity. Hence, the organism would constantly adapt itself to be optimally effective under normal evolutionary conditions of feast and famine, with periodic physical activity to escape predators or find food. Thus, insulin-stimulated mitochondrial biogenesis would be enhanced – a kind of feed forward amplification in the presence of hormetic stimuli.

**Figure 1:**
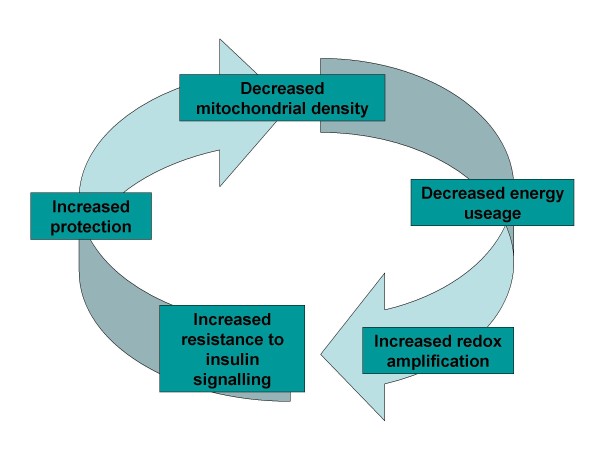
**Insulin resistance protective cycle – the underlying principle for '*redox-thriftiness'***.

As oxidative redox drives growth, we suggest that a 'thrifty' phenotype would probably have a lower mitochondrial density to reduce energy expenditure ('symmorphosis') and enhance mitochondria-mediated ROS amplification; this would both drive insulin resistance and inflammation. During feeding, this reduced mitochondrial density would ensure a rapid amplification of ROS and a potent insulin resistance signal. At low levels, this would ensure storage, but if amplified by infection, it would enhance inflammatory responses (including insulin resistance to ensure energy for the CNS and immune system). Although this phenotype might be altered by acute stressful energy-requiring mito-hormetic stimuli, even during calorie restriction when mitochondrial density may increase, it would be associated with lipid-induced insulin resistance. The concept of '*redox-thriftiness*' is displayed in figure 1.

## Inflammation, a *tipping point*, life expectancy and VAT

We suggest that although optimal in an ancient environment, '*redox-thriftiness' *may lead to a 'redox spiral' in the absence of constant and appropriate hormetic stimuli and the presence of unlimited calories. The ensuing insulin resistance would further inhibit insulin-driven mitochondrial biogenesis, so worsening the spiral. There may, therefore, exist a *thrifty-inflammatory tipping point *when normal physiological thrifty insulin resistance gives way to more generalised inflammatory and pathological insulin resistance [117]. It is therefore likely that the *thrifty-inflammatory tipping point *also has a set point, which is likely to be modulated by both genetics, environment and epigenetics, and would thus be related to metabolic flexibility, and importantly, by the extent of an innate or programmed inflammatory response to oxidative stress.

As aging is associated with increased NFκB activity [118,119], the *tipping point *could also represent the activation of an ancient accelerated aging mechanism to shorten functional longevity and increase population turnover. Accelerated ageing may well have evolved as an evolutionary mechanism against predation, and could also be activated by 'stress' to weed out less fit organisms. In contrast, without predation, the natural state of any species is to develop extreme longevity as this increases reproductive potential [120]. We also suggest that this same mechanism may have become adopted as a mechanism to prevent excessive weight gain.

Atherosclerosis and hypertension are linked via endothelial dysfunction and an imbalance between oxidative and anti-oxidant mechanisms, leading to a vicious inflammatory-oxidative cycle – this is largely driven by moieties that become oxidised, such as LDL. Hence, the development of diabetes accelerates the process through increased oxidative stress induced by hyperglycaemia and insulin resistance [121]. Molecularly this is thought to occur via oxidation of LDL via a number of oxidative and carbonyl mediated mechanisms [122]. Dyslipidaemia, atherosclerosis, the formation of plaques, and ultimately, thrombosis leads to 'atherothrombosis', and is likely to afflict most people [123]. It has been calculated that stochastic damage to elastin in the human vasculature may limit ultimate human life expectancy to about 120 years [124]; thus, any accelerated damage will clearly reduce this. The *tipping point *could have a profound influence on life expectancy, which in humans is largely (but not exclusively), determined by cardiovascular disease: the metabolic syndrome is associated with an earlier than normal onset of many diseases, including renal disease, cancer, osteoporosis, depression and neurodegeneration [125-129].

### The immune system and energy storage; good and bad for the individual

The immune system and the co-evolutionary need to resist famine and infection – the 'thrifty-cytokine' idea [130], which is based on the 'metabolic costs of immunity' [131], may be critical in the metabolic syndrome. Stored energy enables a robust immune response to be mounted, but might lead to a pro-inflammatory state. The 'metabolic syndrome' phenotype is characterised by pathological insulin resistance, dyslipidaemia, hypertension, hypercoagulability, increased VAT and oxidative stress, which shares many similarities (although milder) to what happens in the APR [132,133] and stress response [134]. Indeed, oxidative stress-induced activation of the stress pathways, JNK & p38 MAPK, and the IκBK/NF-κB pathway, may provide a unifying hypothesis to explain T2D [135]. Reduction of JNK1 activity in macrophages can protect against obesity-induced insulin resistance, while JNK1^-/- ^mice are highly resistance to diet induced obesity and appear to have an increased metabolic rate [136,137]. Thus JNK appears to play a central role in obesity and insulin resistance [138].

This therefore presents a paradox; increased activity of JNK is associated with increased lifespan, but in the context of the metabolic syndrome, its activity might be associated with a reduced lifespan. JNK is a ROS-activated kinase and is upregulated by many stresses, and cytokines, and if briefly activated, increases cell survival, however, if continually active, it induces apoptosis. Likewise, NF-κB is also activated by ROS, but in contrast suppresses JNK activity, and thus apoptosis. It may do this, in part, by suppressing ROS by increasing anti-oxidant enzymes [139,140]. This might begin to explain why, although NF-κB activity is increased in the metabolic syndrome, its relationship to insulin resistance may be very tissue specific: it may be acting to aid in cell survival and suppress excessive ROS. This may suggest that at least with regards to insulin signalling, JNK maybe more important than NF-κB. Given the very strong relationship of obesity to insulin resistance, and the macrophage JNK data above, increasing levels of obesity may result in increased pro-inflammatory tone (so increased NF-κB activity), which results in cytokine-induced activation of JNK – the 'redox spiral'.

One source of the inflammation may be 'stressed' adipocytes that become overloaded with fat-attracting macrophages [141]. New data suggest that the number of adipocytes an adult human may be set during childhood/adolescence [142], hence, in adulthood fat capacity may be fixed. This suggests that it is possible to overload the fat storage system. If leptin-deficient mice are engineered to over-express adiponectin (which can suppress NF-κB), they can constantly expand their fat tissue, becoming morbidly obese, but appear to be metabolically healthy with little adipose tissue inflammation and do not become insulin resistant: this ability is associated with increased activity of PPAR γ [143]. PPAR γ is important in adipogenesis and is suppressed by FOXO [144] and in general, it appears that NF-κB and the PPARs may mutually repress each others activity [145-148], which suggests that the PPARs play a significant role in modulating inflammation and insulin resistance, and thus, longevity, as they can be down-regulated by oxidative stress. Insulin can also increase PPAR γ transcription in adipocytes, probably via mTOR [149]. Hence, PPAR γ-driven accumulation of fat is probably protective, but the downside is that it would probably result in an animal too fat to move. Thus, suppression of excessive fat storing activity may be important in limiting size.

It has been long thought that the response to 'stress' can dictate the propensity to a metabolic syndrome phenotype [134]; Cushing's syndrome, in which there is an overproduction of cortisol, generates a very similar phenotype. Cortisol itself results in increased VAT, insulin resistance, hepatic gluconeogenesis and lipogenesis, increased lipolysis and reduced insulin output. Both the sympathetic nervous system (SNS) and hypothalamic pituitary adrenal (HPA) axis are more active in obesity and the metabolic syndrome. Cortisol also positively modulates 24 hour leptin production, and at low concentrations, can enhance insulin's actions, rather than inhibiting them [150]. The increased activity of the SNS and HPA may also be as a mechanism to prevent excessive weight gain, and is associated with insulin resistance, and may be one of the actions of leptin [151,152].

It is therefore possible that it is the response to stress itself that is important, and as previously mentioned, this might represent a 'weeding out' mechanism for less fit organisms. However, glucocorticoid release, under normal circumstances, prepares the body to meet increased metabolic demands – for instance, fasting or exercise, or even perceived stress [153]. Thus, although the metabolic syndrome can be partly explained by increased activity of the SNS and HPA, it is also likely that it might represent a response to a more fundamental stress. Corticosteroids are strongly anti-inflammatory and can both induce endocannabinoid release (possibly redirecting arachidonic metabolism towards anti-inflammatory mediators, rather than inflammatory prostaglandins) [154], and in some tissues, can induce mitochondrial biogenesis [113,114]. This might suggest why the number of fat cells may eventually become fixed: it is a size limiting mechanism – as fat cells become more stressed, they start to drive an anorexic response – which may be very similar to the metabolic syndrome. The above suggest that storing energy is essential to mount an immune response, but this same mechanism may also start to drive a response to limit size using inflammation.

### Origins of the dyslipidaemia; inflammation

Acute injury or infection activates the APR, which is associated with release of acute phase proteins, hepatic gluconeogenesis, hyperlipidaemia and insulin resistance [155]. The process is driven by cytokines and is also associated with decreased fatty acid oxidation, increased fatty acid synthesis and triglyceride formation, as well adipose lipolysis [156]. Likewise, the metabolic syndrome is associated with decreased HDL-c and increased triglycerides, as well as changes towards more inflammatory (acute phase) apolipoproteins, with reduced particle size and the presence of oxidised lipoproteins. It is thus associated with a very similar inflammatory lipid profile [157]. VAT is metabolically very active, and is sensitive to the lipolytic effect of catecholamines, but insulin resistant – it appears to be in a permanent lipolytic mode. This results in high levels of FFA being delivered to the liver and an increase in hepatic lipase activity; this also decreases lipoprotein particle size. Critically, as the size of adipocytes increases, so does the production of lipoprotein lipase (LPL) and cholesterol ester transfer protein (CETP), as well as angiotensinogen, PAI-1, IL-6 and TNFα. Insulin and cortisol increase LPL production – which may explain why activation of the HPA axis may result in increased VAT [158].

It is now widely acknowledged that atherogenesis is related to an inflammatory lipid profile, and that the lipid carrying system is also part of the immune system. For instance, although HDL can via apolipoprotein A-1 have a vital role in reverse cholesterol transport and reduce oxidative stress, HDL can also demonstrate a more pro-inflammatory nature, as it can carry many APR components [156,159]. Thus, the dyslipidaemia and insulin resistance in the metabolic syndrome have all the hallmarks of being driven by inflammation, which itself, is most likely triggered by oxidative stress.

### A thrifty-inflammatory *tipping point *and a function for VAT?

Excessive substrate levels, inefficient autophagy and stress signalling would simply overwhelm many cells. This would explain the increased endoplasmic reticulum stress found in obesity and diabetes [160], which leads to inflammation [161]. This might have the effect of worsening the lipotoxicity by inhibiting the PPARs, in particular, PPAR γ, so reducing the capacity to increase pre-adipocyte proliferation. In effect, rising inflammatory tone might lead to a reduced capacity to metabolise and store fat safely, as it might lead to insulin resistance in adipose tissue, resulting in lipolysis.

Thrifty insulin resistance could be determined by reduced mitochondrial function and '*redox-thriftiness'*: this ensures both energy storage, resistance to excessive redox signalling and, quite possibly, a hair-trigger inflammatory response. As fat mass increases, there is a gradual improvement in the ability to mount a strong immune response, however, if it is not offset by mitohormetic stimuli, then it is possible that the innate immune system and the HPA/SNS become activated. This might initially have the effect of mildly increasing insulin resistance still further. However, in combination with excessive calories and rising inflammatory signals, many cells become "stressed" and start to inhibit essential functions like mitochondrial biogenesis and fat storage. At this point a vicious feed-forward loop is initiated.

**Figure 2:**
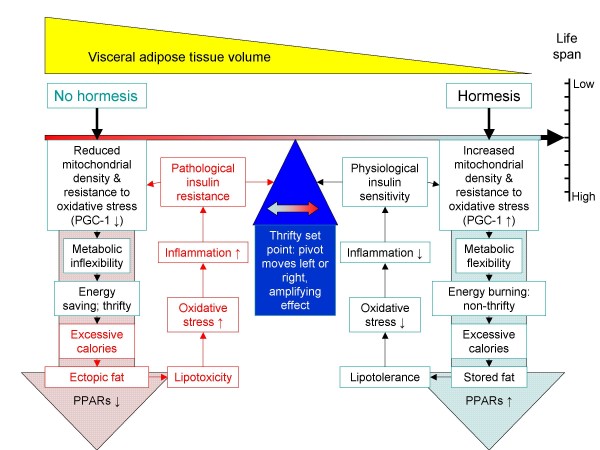
**The *tipping point *and the metabolic syndrome**. Without hormesis, and in the presence of excess calories, VAT can build up, which is associated with excessive ectopic fat due to metabolic inflexibility. This is associated with rising oxidative stress and a shift from thrifty to inflammatory insulin resistance; this results in the metabolic syndrome and an accelerated ageing phenotype.

Thus thrifty insulin resistance may develop into inflammation-driven insulin resistance; this itself may be a mechanism to prevent excessive weight gain. Insulin resistance in adipocytes, in particular, those in VAT, would lead to increased lipolysis – a symptom of the adipocyte becoming increasingly insulin resistant. The increased activity of the HPA axis, with rising levels of corticosteroids, might even act to accelerate fat burning in adipocytes. This data may then shed light on a function for VAT: it modulates maximum fat storage and life expectancy. New data suggest that indeed, VAT can modify life expectancy – it's removal extends lifespan [162]. Both calorie restriction and exercise result in a rapid depletion of VAT; this may support the hypothesis of Freedland who suggested that there is a critical visceral adipose tissue threshold (CVATT) [163]. Figure 2 summarises this concept: without hormesis, metabolic flexibility decreases and in concert with excessive calories, ectopic fat is deposited, in particular, in the visceral region – this drives an inflammatory response that may well act to prevent excessive weight gain, but it will also shorten lifespan. In contrast, in the presence of hormetic stimuli, this is much less likely to happen – as any excess calories may be directed to other fat stores (such as subcutaneous fat) or burnt off.

## Modulation of the *tipping point*

It is likely that the *tipping point *may be determined by many positive hormetic and negative inflammatory factors, which in turn, modulate the '*redox-thriftiness' *set point and metabolic flexibility. Physical activity is probably one of the strongest positive stressors, as is fasting: alternate day calorie restriction (fasting) can invoke many of the beneficial effects of calorie restriction in both animals and humans [164-166]. Inflammation is clearly a negative regulator (increases oxidative stress and induces insulin resistance), and although beyond the scope of this paper to fully review, it is likely that many infections, such as hepatitis C which are associated with increased rates of T2D [167], could profoundly effect the tipping point.

Dietary composition may also strongly influence it: diets high in saturated fat tend to be detrimental, whereas diets high in unsaturated/polyunsaturated fats may be more healthy [168-170]. In stone age times, the ratio of polyunsaturated to saturated fat (P/S) in the diet was probably nearer 1.0 [171,172]. In comparison, dietary studies in the late 1970 s and early 1980 s indicated that the P/S ratio was 0.20 and 0.35 in Australia and Finland, respectively [173,174]. This probably means that humans have evolved (to varying degrees, depending on environment), to be dependent on a dietary-, exercise-, temperature-, and fasting-induced levels of hormesis. This would explain why many clinical trials of simple anti-oxidants, such as vitamin E, have failed [175]; simply blocking free radicals, because of the their role in redox, may reduce intracellular preconditioning effects. In contrast, some polyphenols, such as resveratrol do actually appear to have a benefit as they can induce mitochondrial biogenesis. In this regards, even polyunsaturated fats could be viewed as 'hormetic'. This also extend to other compounds, including some pharmaceuticals, such as statins, metformin, or even alcohol.

### *Tipping point*: polyunsaturated hormesis?

PUFA may be potentially hormetic due to their double bonds; the greater the degree of unsaturation, the greater the potential for auto-oxidation [176]. Thus, the observation that the membranes of mitochondria generally have a lower unsaturation index than other membranes in the cell (containing more MUFA, but less PUFA), suggests reduced susceptibility to membrane damage [177]. Oxidation of highly unsaturated fats leads to reactive molecules, such as malondialdehyde (MDA) [178]. Excessive omega-6 PUFA can instigate mitochondrial nitrosative damage [179], while omega-3 PUFA, but not MUFA, or saturated fats, can induce the release of mitochondrial calcium [180]. Rats fed for life a diet very high in PUFA have a shorter lifespan, but can be protected by coenzyme Q_10 _supplementation [181]. In a model of breast cancer, feeding pre-pubescent rats controlled levels of n-3 PUFA was protective and associated with reduced DNA damage, whereas feeding pre-pubescent rats with a high level of n-3 PUFA was associated with an increase in cancer and DNA damage [182].

Is there evidence that unsaturated fats invoke a protective response? Unsaturated fats are better ligands for PPAR γ and δ than saturated fats; PPAR α is less selective and more promiscuous [183]. This bias may also be evident for the uncoupling proteins (UCPs), with unsaturated fats, in particular PUFAs, being more potent UCP activators than saturated fats [184,185]. One function of the UCPS may be to transport out potentially damaging lipid peroxides from mitochondria, so reducing oxidative stress. The mechanism is thought to involve superoxide activation via a free radical chain reaction that forms reactive aldehydes, such as hydroxynonenal (HNE) derived from omega-6 PUFAs, or hydroxyhexenal, from omega-3 PUFAs, which are particularly susceptible to peroxide damage [186].

Lipotoxicity (or at least, a switch to lipid metabolism) is an important contributor to insulin resistance. However, this may be dependent on the type of fatty acid. For example, palmitate, but not unsaturated fatty acids can induce myotube IL-6 production [187], while mice over-expressing muscle UCP-1, despite having high levels intramyocellular fat, are still insulin sensitive [188]. Certainly, unsaturated fat can undergo futile cycling, whereas saturated fat does not appear to and can lead to lipotoxicity [189]. Reduced functioning of UCP-3 could lead to mitochondrial lipotoxicity, reduced oxidative capacity and could contribute to ageing and type 2 diabetes [190]. Increased activity of UCP-2 can protect against obesity, while decreased activity is associated with type 2 diabetes [191]. Certainly, there is evidence that PGC-1α, which can modulate UCP transcription, is down regulated in type 2 diabetes [192]. Their role in fatty acid metabolism is suggested by the observation that the activity of UCPs is increased during starvation and by a ketogenic diet [193-195].

Different fatty acids have different insulinotropic capacity and are critical for glucose-stimulated insulin secretion (GSIS). It is dictated by the degree of unsaturation and chain length – increasing with chain length, but decreasing with degree of unsaturation [196]. Increased PPAR γ activity suppresses GSIS by upregulating UCP-2 [197], while PPAR α has been found to be involved in the pancreatic adaptation to fasting by also upregulating UCP-2 [198]. This could be indicative of hormesis. Combined with the well described ability of PPAR γ to improve glucose dispersal, which is now also being described for PPAR δ [199], as well as PPAR α [200,201], it is likely that the type of fatty acid can modulate both ends of the insulin axis. For instance, unsaturated fats may reduce the stress on the insulin axis by maintaining insulin sensitivity, but reducing GSIS. This may well suggest improved mitochondrial function and a hormetic effect: unsaturated fatty acids, in the pancreas, would upregulate anti-oxidant systems (including mitochondrial biogenesis) that would reduce the glucose-induced ROS signal from the mitochondrium. In the periphery, this would tend act to maintain insulin sensitivity by damping down stress-signalling that would other inhibit insulin signalling function.

### *Tipping point*: saturated fats and inflammation

Saturated fats have a higher melting point than unsaturated fats, so they make membranes less fluid. The anti-inflammatory action of glucocorticoids is thought to partly occur by decreasing the saturated fatty acid content, while increasing the unsaturated content of lipid rafts, so increasing membrane fluidity [202]. Saturated fats are also a major component of bacterial cell walls, and may activate the innate immune system via TLRs (toll-like receptors), whereas unsaturated fats, in particular those of the omega-3 series, inhibit TLR activation [203]. Mice lacking TLR-4 are substantially protected from lipid-induced insulin resistance [204]. Lipid rafts are key in immune and insulin signalling, and as suggested above, their function can be altered by either cholesterol depletion or by increasing the content of unsaturated fatty acids – both of which have an anti-inflammatory effect [205]. TLRs also signal through lipid rafts, which are an important site of ceramide release. Ceramide is a critical part of the ancient sphingomyelin stress signally pathway [206] and is associated with the development of insulin resistance [207].

Saturated fat is known to induce athrogenic hyperlipidaemia, a process involving hepatic PGC-1β and SREBP [208]. Saturated fat is also less effective than unsaturated fat at stimulating the incretin glucagon-like peptide 1 (GLP-1) from the gut [209]. The biological activities of GLP-1 include stimulation of GSIS and insulin biosynthesis, inhibition of glucagon secretion and gastric emptying, and inhibition of food intake.

Altogether, this does suggest that a diet high in saturated fat is more likely to induce insulin resistance. Data does tend to support the notion that reverting to diet more like that of our ancestors by reducing saturated fat, but increasing unsaturated fats, with a high omega-3/omega-6 ratio may improve insulin sensitivity [210]. Certainly, a diet high in saturated fat can lead to obesity [211], while epidemiological data does imply that replacing saturated fat with unsaturated fat can improve many symptoms of the metabolic syndrome, including insulin sensitivity [212]. The above suggest that excessive saturated fat may be non-hormetic and inflammatory.

### *Tipping point*: the role of anti-inflammatory lipids

Malcher-Lopes and colleagues suggest that the glucocorticoid-induced release of 2-AG and anandamide (AEA) is part of a mechanism to divert arachidonic acid from inflammatory mediators (the prostaglandins, involving COX-2), to anti-inflammatory mediators (the endocannabinoids) and a protective profile [154]. Both endocannabiniods and novel docosanoids are neuroprotective following ischaemia-reperfusion injury [213,214]. Interestingly, hypoxic brain injury induces a rise in mitochondrial biogenesis [215].

Endocannabinoids are released on demand, generally by stressful stimuli, for instance, by stressed adipocytes – which, it has been suggested, may be part of the cause of obesity and the metabolic syndrome due to overactivity of the endocannabinoid system via a feed-forward mechanism [216]. This apparent dysfunction in obesity has led to the development of CB-1 receptor antagonists, such as rimonabant, for the treatment of obesity (and its sequalae) [217]. Although these drugs do induce a degree of weight loss and reduce symptoms of the metabolic syndrome, their long term use is limited due to CNS side effects, suggesting alternative approaches may be needed – such as partial agonism [216]. Rimonabant does reduce pro-inflammatory and pro-thrombotic markers in diabetic Zucker rats, suggesting a broad anti-inflammatory action [218], and it does improve insulin sensitivity in some tissues; however it also enhances HPA activity in food-deprived Zucker rats [219] and increases production of corticosteroids [220]. This suggests it is activating a stress response.

In adipocytes, 2-AG may improve insulin sensitivity, while rimonabant reduces it [221]. In muscle, CB-1 receptors may, via ERK and P38 kinase (but not NF-κB or JNK), inhibit insulin action [222]. At the cellular level, rimonabant decreases the fat content of 'obese' adipocytes by increasing lipolysis, futile cycling and fatty acid oxidation, which is supported by the transcriptional profile [223]. It also appears to increase mitochondrial biogenesis in white adipocytes, a process mirrored in CB-1 knockout mice [224]. In light of this, we suggest that rimonabant, via increased adipocyte insulin resistance, enhances lipolysis and in concert with raised levels of corticosteroids, stimulates adipocyte mitochondrial biogenesis. It thus may exaggerate a stress response; this may be driven by increased CNS stress. Although the appetite suppressing effects of rimonabant are rapidly lost, clinical trials show a clear increase in CNS side effects, which has led to a high discontinuation rate [225].

Rimonabant may therefore be inducing increased energy turnover by accelerating the previously described adipose-inflammation-stress weight prevention mechanism. But at what cost? CB-1 receptor knock out mice, although lean and resistant to a high fat diet, have a reduced life expectancy [226]. Their transcriptional profile is also similar to that induced by rimonabant [223]. This might suggest that very long term and potent inhibition of the CB-1 receptor may be detrimental. Furthermore, CB-1 receptors may activate AMPK in the brain and heart, but suppress it in the liver and adipose tissue [227]. The above proposed mitochondrial biogenic mechanism of rimonabant in white adipose tissue may suggest that at least in the heart and brain, it may actually reduce mitochondrial biogenesis. It could also in the long term lead to more generalised adipose tissue dysfunction and exhaustion.

With regards the *tipping point*, it would appear that the inflammatory insulin resistance profile superimposes over the thrifty insulin resistance profile, resulting in the adipocyte becoming insulin resistant and amplifying the inflammatory metabolic profile. This may well invoke a thermogenic energy wasting response, which is negatively regulated by increased endocannabinoid release. Furthermore, as endocannabinoids are now being shown to be PPAR agonists [228], then they may well may increase adipogenesis. Thus, endocannabinoids could be exerting anti-inflammatory and adipogenic actions in VAT, which may actually be protective. Teleologically, insulin resistance in most organs is protective, but in adipose tissue, it may be essential to maintain insulin sensitivity to store fat until the organism gets too fat; then the development of adipocyte insulin resistance prevents excessive weight gain – but this may come at a price.

### *Tipping point*: glucose as an inflammatory signal?

Restricting glucose availability to *C. elegans *results in oxidative stress and induces mitochondrial biogenesis and improved longevity [101]. High levels of glucose result in increased mitochondrial superoxide generation and ROS [229], which is also an inflammatory signal [230]. Hyperglycaemia can activate the inflammatory system via advanced glycation end-products (AGE), which are known to increase NFκB activity [231], leading to localised and systemic insulin resistance. Furthermore, glucose can also induce the release of APR reactants from adipose tissue [232]. Glucose could, therefore, be viewed as an inflammatory mediator, which would support Dandona's concept that insulin can be viewed as anti-inflammatory [233] and probably has immunomodulatory functions. Hence, excessive levels of high glycaemic index carbohydrates could not only result in a large amount of saturated fat being created (as glycogen stores become saturated), but if the pancreas was unable to cope, hyperglycaemia. Certainly, there is good evidence that high carbohydrate diets are more likely to result in the metabolic syndrome, which is supportable by basic biochemistry [234]. It is thus possible that hyperglycaemia could actually be seen as inflammatory and be the final 'coup de gras' that triggers feed-forward inflammation as the pancreas decompensates. It could, again, also be viewed as a mechanism to prevent excessive adiposity.

### *Tipping point*: polyphenol 'xenoergohormesis'

Many non-nutritional components of plants modify transcription, the most well known are the isoflavones (PPARs), epigallocatechin gallate (EGCG, inhibits the proteosome), hyperforin (activates cytochrome P450) and resveratrol (activates sirtuins) [235]. It has been known for some time that polyphenols, such as EGCG, can inhibit NF-κB activation [236], which would lead to reduced inflammatory response, as well as insulin signaling [237]. Some polyphenols can also mimic the longevity effects of sirtuin activation, which are known to be critical in calorie-restriction induced longevity [238]. This sirtuin modulating ability has now also been shown for several isoflavones, and is associated with mitochondrial biogenesis [99]. It has also been shown that activation of the retinoid × receptor (RXR), the obligate dimeric partner of PPAR, can induce thermogenesis [239]. Plus, phytanic acid, a naturally occurring component of many foods, can also activate both PPARs and RXRs [240], while activation of RXR by rexinoids can improve insulin sensitivity [241]. Retinoic acid can also suppress NF-kB activity, which is associated with a switch from a Th1 to a Th2 response [242].

It is thought that most polyphenols are secondary metabolites involved in plant-defence against stressors such as ultraviolet light or insects, and many are toxicants that can suppress growth and can inhibit many aspects of arachidonic-based inflammatory pathways [243]. However, many anti-oxidant components in plants can also be viewed as part of the plant redox-signalling system; if a plant is stressed, these components are upregulated to suppress excessive ROS-driven damage, for instance, ascorbic acid, α-tocopherol and reduced glutathione. However, they could also be viewed as a means of suppressing excessive redox signalling [244]. Animals and plants also share a very high degree of sequence homology between the ERK pathways [245]. Thus the observation that many polyphenols can modulate kinase pathways in animal cells [246,247], including AMPK [248], is relevant.

Critically, polyphenols appear to have pleiotropic actions, and can directly modulate mitochondrial function, often resulting in increased ROS production. For instance, several polyphenols (including resveratrol) can inhibit mitochondrial proton F0F1-ATPase/ATP synthase [249], while tetrahydrocannabinol (THC) has been found to inhibit complexes of the mitochondrial electron transport chain [250]. However, they may be acting in other ways as well. For instance, resveratrol can activate MAPK inducing eNOS; one way it could be doing this is via activation of the oestrogen receptor [251,252] – activation of the oestrogen receptor has been shown recently to modulate mitochondrial function and decrease superoxide production [253]. Interestingly, resveratrol has also been shown to inhibit HNE activation of the JNK pathway [254], as well as insulin signalling to Akt and MAPK – which was not dependent on sirtuins [255].

**Figure 3:**
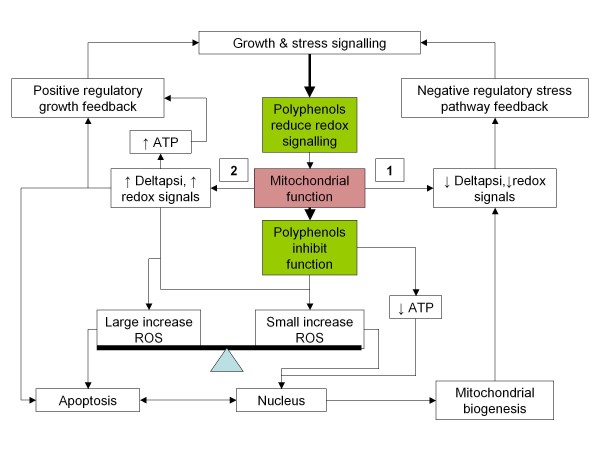
**How polyphenols might work**. Blocking or modulating growth and stress signalling reduces growth drive and redox stress, so reducing need for activation of 'growth' stress inhibitory pathways (e.g. JNK). Mildly inhibiting mitochondrial function may decrease ATP and increase ROS, which is a strong stimulus for mitochondrial biogenesis (it is a hormetic signal), resulting in an enhanced anti-inflammatory/ROS cellular phenotype (1). However, excessive inhibition of this pathway may induce apoptosis in some cell types (2). This paradigm might follow the general evolutionary function of these polyphenols in plants: improved resistance to stress and pathogens.

In light of the data, we propose that it is possible that some polyphenols may be simultaneously capable of modulating membrane based redox signalling, while inhibiting mitochondrial function: this would have the effect of reducing stress signalling (e.g. derived from inflammatory signalling), but increasing mitochondrial ROS ('oxidative preconditioning'), while reducing ATP production – a potent mitochondrial biogenic signal. The recent observation that MAPK also locate to the mitochondrium may be important in this regard [83]. In essence, they may reduce the ATP/ROS ratio, which is a powerful hormetic signal. From the point of view of a plant, this is both a potent stress signal and an effective way (at high doses), of inhibiting pathogens. Figure 3 outlines the hypothesis.

This could explain the observation that resveratrol can offset the life-shortening effects of a high fat diet. In mice, resveratrol is associated with increased activity of PGC-1α and AMPK, as well as improved insulin sensitivity [256]. Certainly, there is good evidence that many small molecule activators of the sirtuins, such as resveratrol, can extend life in *C. elegans *and *D. Melanogaster *[238], as well as in fish [257]; this may support the 'xenohormesis' theory cross-species signalling mechanism [258]. In addition, the concept of 'exercise mimetics' has been suggested by Narkar and colleagues [259]: this involves pathways and factors, such as PPAR δ and AMPK, which are known to be involved in modulation of PGC-1α. The endurance improving effect of resveratrol [260] might suggest that the 'xenohormesis' idea could be extended to the concept of 'xenoergohormesis', where the eating of plant polyphenols optimally modulates the exercise capacity of an animal when food is available.

### *Tipping point*: accidental hormetic agents and '*redox-thriftiness*'

Other than the polyphenols discussed before, several marketed pharmaceuticals (and other compounds) can reduce metabolic syndrome markers and may exhibit hormetic effects. One of the oldest may be metformin. It is still first line therapy after lifestyle change for the treatment of type 2 diabetes; it is also one of the few to actually induce weight loss [261]. It has now been proposed that its mode of action may involve inhibition of mitochondrial complex 1, which increases ROS, and in combination with NO, increases peroxynitrite which activates AMPK [262], which then upregulates PGC-1α [263]. This strongly suggests it is hormetic, and it does improve insulin sensitivity.

Another class of well used drugs are the statins. They can improve the dyslipidaemia of the metabolic syndrome, and have been shown to reduce the associated inflammation and oxidative stress [264]; they also reduce blood pressure slightly [265]. With regards insulin resistance, the data is mixed [266-268]. One of their main side effects is myopathy. One explanation is that they might increase oxidative stress by decreasing production of mitochondrial coenzyme Q_10_, a potent anti-oxidant [122,269]. In addition, they can also directly induce mitochondrial dysfunction by inhibiting oxidative phosphorylation and uncoupling, a property they share with fibrates and glitazones [270]. However, they can also induce a preconditioning effect by stimulation of NO and carbon monoxide and can can activate AMPK [271-274]. It is therefore possible that although their benefits are limited by inducing mitochondrial dysfunction, they may also be hormetic. Thus, they may well display a bimodal effect: in patients who are already likely to have severely compromised redox pathways, they may well be less effective. But in others, they may induce just enough oxidative stress to be protective. This may well suggest an alternative mode of action for the statins: it is not the cholesterol lowering in the blood, per se, that is important, but actually their hormetic effect.

Finally, one other well observed drug is alcohol. Across society its effects appear to follow a 'U'-shaped curved, being beneficial at lower doses, while becoming toxic at higher doses. One of its effects is to both increase both the quantity and protective qualities of HDL-c, perhaps via increased lipidation [275]. Alcohol is also well described to induce oxidative stress, principally via a mitochondrial mechanism that can result in mitochondrial dysfunction [276]. Too much alcohol can cause certainly insulin resistance by damaging the liver [277], in contrast, there is some data that small amounts of alcohol can be associated with improved insulin sensitivity in healthy adults [278,279]; however, the true extent of this beneficial effect may be partly confounded by body composition and lifestyle [280,281]. Although there are clearly many factors which may obscure an effect, the above does suggest that alcohol could, at the right doses, have a hormetic effect.

### *Tipping point*: epigenotypes, hormesis and metabolic flexibility

It has been suggested that as feast and famine were the normal state of affairs during evolution, the thrifty trait has become genetically canalised and is thus a robust characteristic of all life. However, it is modifiable and its expression is thus a combination of both the genotype and the environmentally induced phenotype – or the 'epigenotype' [16]. One key factor in modifying the epigenotype must be hormesis, in particular, the metabolic flexibility epigenotype epitomised by mitochondrial function.

**Figure 4:**
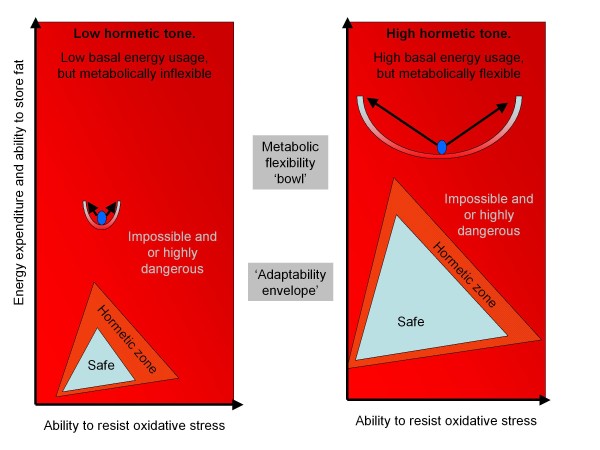
**Depictions of metabolic flexibility**. The metabolic flexibility 'bowl' and metabolic 'adaptability envelope'.

With little or no hormetic stimuli, there is likely to be a gradual reduction in mitochondrial density and a commensurate decrease in both metabolic flexibility and resistance to oxidative stress. The benefit is that this reduces the need for energy. In effect, economy of design, or symmorphosis, reduces metabolism and structure to the minimal needed. Two ways of viewing this are depicted in figure 4. The first is the metabolic flexibility bowl, which represents the epigenotype canal. Without hormesis, the bowl becomes narrower, and the sides shorter; it doesn't take much to push the organism to the edge – but this may provide a powerful signal to adapt. However, too much, and the organism will not survive or become severely compromised. This is also depicted by the adaptability envelope idea (similar to a flight envelope for an aircraft), whereby there is a safe zone, a zone which is dangerous, but stimulates adaptation – but then a dangerous no-go zone. For instance, fasting would improve resistance to oxidative stress and the ability to store fat safely (more, smaller adipocytes), whereas both physical activity and cold would induce mechanisms to burn fat safely (e.g. mitochondrial biogenesis), as well as also improving the potential to store energy. Under normal circumstances, all of these would combine to ensure optimum adaptability. However, without these, continual calorie intake would exceed the ability of the organism to deal with the extra lipids beyond its hormetic adaptability zone, resulting in excessive oxidative stress and inflammation. This would push the organism past the tipping point and either out of the bowl, or into the no-go area. This could then result in the accelerate aging phenotype.

## *Redox-thriftiness*, insulin resistance and evolutionary suicide

**Figure 5:**
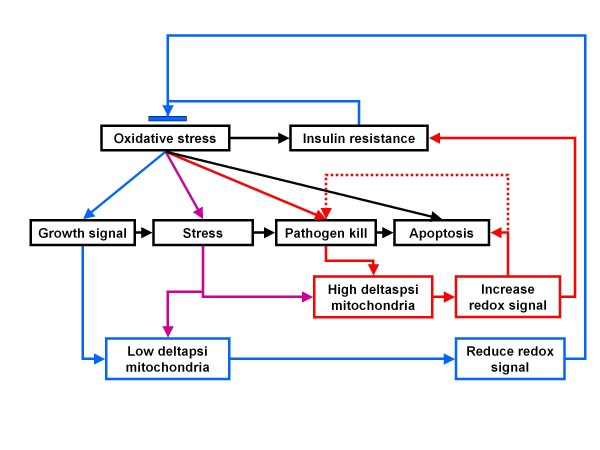
**Oxidative stress increases insulin resistance**. Oxidative stress increases insulin resistance, which is a feedback mechanism to reduce oxidative stress that is modulated by mitochondrial function.

The concept of '*redox-thriftiness' *suggests that insulin resistance is induced by oxidative stress and is thus a protective mechanism. Hence, the ability to resist oxidative stress is associated with insulin sensitivity. As mitochondria are critical in determining resistance to oxidative stress, then insulin resistance may be determined by the amount of ATP produced by mitochondria in relation to their ROS output; having a high density of low potential mitochondria is probably a mechanism to reduce redox signalling and thus, oxidative stress (figure 5). However, whether insulin resistance is viewed as friend or foe depends on whether it is seen from the viewpoint of the cell, an organ, the individual organism, or the species: within '*redox-thriftiness' *may lie a higher order mechanism to improve the fitness of the species at the expense of the individual, although, paradoxically, it improves the survival of the cell or organism in the short term.

### From the selfish cell to the selfish brain

The brain is almost totally dependent on glucose: although it constitutes only 2% of the body mass, its metabolism accounts for 50% of total body glucose utilization. Although the brain does not require insulin to take up glucose, insulin receptors are found in many areas of the brain and are vital for normal function. Thus, insulin resistance in the brain could have an impact on the origins of the metabolic syndrome and the propensity to increase obesity [282]. In obesity, the brain becomes insulin resistant and can have too much glucose, which is associated with accelerated brain aging and may involve NO-induced oxidative damage to neuronal mitochondria [283,284]. However, both starvation and triglycerides reverse obesity-induced suppression of insulin transport across the BBB [285].

The 'selfish-brain' brain hypothesis in relation to the metabolic syndrome posits that insulin resistance and activation of the SNS/HPA are part of a normal system to maintain a set point to maintain glucose to the brain. The brain uses glucose via a localised 'on demand' system, but as circulating glucose would rapidly run out, it also ensures an 'on request' allocation system to ensure supply, which may also be part of the stress reponse system. When food intake is low (resulting in mild stress), glucose supply is maintained to the brain via gluconeogenesis, insulin resistance and suppression of insulin release. When food is plentiful, the stress system relaxes and the body becomes insulin sensitive and fat stores are increased until both leptin and insulin levels suppress energy intake. However, if food is excessive, then insulin and leptin signals rise, activating the SNS, suppressing appetite. If over-eating continues, the organism gets further away from an ideal body weight set point, resulting in a continual mild activation of the stress system (as it attempts to compensate): it is well described that untreated diabetes does result in weight loss [152]. Indeed, obese patients generally have a higher basal metabolic rate (BMR) [151], which does support this hypothesis.

We would suggest that this hypothesis can be integrated with the '*redox-thriftiness' *concept to encompass the 'selfish cell'. As it is likely that glucose readily diffuses across the BBB, and that GLUT-1 and GLUT-3 in the brain are inversely related to glucose levels [286], hyperglycaemia is clearly as potentially dangerous to neurons as it is to other cells. Insulin-induced vasodilatation signals through the Pi3K/Akt pathway. Thus, endothelial insulin resistance is probably associated with excessive insulin in combination with many inflammatory factors, such as oxidised LDL or hyperglycaemia [287]. Hence, BBB insulin resistance could be viewed as a brain protective mechanism. Certainly, there is data to suggest that a degree of reduced insulin signalling in the brain is also associated with an increase in lifespan [288]. Taken together, a mild degree of CNS insulin resistance may also be protective, and would fit the '*redox-thriftiness' *hypothesis. This might explain why the set point may move to a higher body weight: as the brain receives increasing signals to activate the SNS via leptin and insulin, it becomes mildly resistant – which ensures continual positive energy deposition. Hence, the selfish cell concept would help to explain the concept of the 'selfish brain'. One corollary of this would be the development of insulin resistance in adipose tissue, which could also be viewed as a mechanism to prevent excessive weight gain. In this respect, the concomitant increase in HPA activity would not only drive lipolysis via sympathetic innervation, but quite possibly, increased mitochondrial biogenesis – which would both enhance energy usage and protect against lipotoxicity.

### From the selfish brain to the selfish species

Is the accelerated aging phenotype associated with the metabolic syndrome simply a by-product of an unnatural evolutionary situation, or could it have an adopted function, such as a mechanism to increase population turnover via reducing life expectancy in times of plenty? For instance, it might partly explain one widely accepted evolutionary lifespan hypothesis called the 'disposable soma theory'. This is driven by the balance of energy allocation between reproduction and somatic maintenance (e.g. resistance to oxidative stress and repairing the genome). During times of hardship, somatic maintenance improves Darwinian fitness (longer breeding cycle resulting from improved resistance to oxidative stress), whereas in times of plenty, resistance to predation and rapid breeding become more important. This might be related to a decrease in growth hormone releasing hormone from the hypothalamus in response to starvation, resulting in decreased activity of the insulin/insulin-like growth factor axis [289]. The origins of this may well be very ancient indeed, and go right back to the development of unicellular organism apoptosis – as demonstrated by yeast and many phytoplankton.

It is thought that mitochondria are descendents of a group of bacteria (α-proteobacteria) that contain metacaspases. A critical determinate of cell death is oxidative stress [290,291]. Mitochondria may well undergo a form of programmed death, 'mitoptosis', which can drive cell death, 'apoptosis', which in turn can drive accelerated senescence of the entire organism or 'phenoptosis' [292]. Thus, '*redox-thriftiness' *and insulin resistance can be viewed as a mitochondrially-associated mechanism to resist oxidative stress, which is modulated by the environment to ensure survival of the species. Optimal fitness thus comes when a species lives in its hormetic zone; many humans now patently live well outside it – as their environment is far to benign [80]. A disturbing possibility is that that it may be an example of the beginnings of a man-made evolutionary suicide [293,294]. For example, some countries in the Gulf have obesity rates of 30–50%, with diabetes rates approaching 20–30% or more in some places [295-297]. The discovery of oil in this region in the last 60 years has resulted in an unprecedented explosion of wealth in only 2–3 generations – well within epigenetic 'memory' of a tougher time. Virtually all hormetic stimuli have probably been removed. As the median age in these countries is relatively low, it could begin to have a serious impact on the average life expectancy. Developing diabetes at a young age can reduce life expectancy by at least 14 years [298]. However, morbidity sets in long before frank diabetes develops. For instance, excessive insulin resistance and diabetes are both associated with a decrease in cognitive ability [299,300], while the metabolic syndrome is associated with a significant decrease in a broad range of sexual function metrics in both sexes [301]. In short, populations in the Gulf are now well out side their 'metabolic flexibility bowls' and 'adaptability envelopes'.

## Conclusion: the "Goldilocks hormetic zone" and the LIMIT-AGE syndrome

Mild oxidative stress and mitochondrial biogenesis lead to optimal functioning, metabolic flexibility and an improved ability to resist toxic oxidative stress. Hence, modern man needs to live in a hormetic zone that stimulates optimal functioning, a sort of "Goldilocks hormetic zone" – neither too severe, or too easy.

Physical activity and calorie restriction are two potent mechanisms hormetic mechanisms, but emerging data now also support some polyphenols as having hormetic effects. In particular, molecules that result in activation of AMPK, PPAR δ and PGC-1α [259,260]. Certainly, there are other multiple hormetic stimuli that might have a longevity inducing effect, including heat stress and dehydration; the commonality being activation of common stress pathways [302,303]. It is also possible that some pharmaceuticals may have hormetic effects. Thus, our view of some diseases may well need to change: a lack of hormesis reduces our ability to withstand excessive calories due to metabolic inflexibility, leading to the increased expression of maladies ranging from atherosclerosis, to diabetes, to cancer. This suggests zero hormesis is the most dangerous of all, and that any additional hormesis will bring about some benefit. This may well be epitomised by the high risk of a sedentary lifestyle, and the benefit of even some exercise – even if taken up in later age [304,305].

In conclusion, '*redox-thriftiness' *and insulin resistance may be part of highly conserved survival mechanism. In some circumstances, it may also lead to the metabolic syndrome, which may be better described as the LIMIT-AGE concept. *Redox-thriftiness *is tightly linked into mitochondrial function and thus must be modulated by hormetic stimuli. This explains why exercise and nutritional 'stressors' are potent treatments for the metabolic syndrome. The hypothesis may also begin to explain some failures, and successes, of modern day therapeutic treatments.

## Abbreviations

2-AG: 2-arachidonylglycerol; AEA: anandamide (N-arachidonoyl-ethanolamide); AgRP: agouti-related peptide; APR: acute phase response; AMPK: adenosine monophosphate kinase; BAT: brown adipose tissue; BBB: blood-brain barrier; BMI: body mass index; BPI: bactericidal/permeability-increasing protein; CB-1: cannabinoid receptor 1; CBD: cannabidiol; CREB: cAMP response element binding protein; DGL: diacylglycerol lipase; ECS: endocannabinoid system; EGCG: epigallocatechin gallate; ERK: extracellular signal-regulated kinase; ETC: electron transport chain; FPG: fasting plasma glucose; FOXO: forkhead box class-O 1; GLP-1: glucagon-like peptide 1; GSIS: glucose-stimulated insulin secretion; HO: haem oxygenase; HIF-1α: hypoxia inducible factor-1α; HPA: hypothalamic pituitary adrenal; HNE: hydroxynonenal; IGF-1: insulin-like growth factor 1; IMTG: intra-myocellular triglyceride; JNK: c-jun N-terminal kinase; LPS: lipopolysaccharide; MDA: malondialdehyde; MAPK: mitogen activated protein kinase; MPTP: mitochondrial permeability transition pore; NAFLD: non-alcoholic fatty liver disease; NF-κB: nuclear factor κB; NPY: neuropeptide Y; O-GlcNAc: O-linked-N-acetylglucosamine; OEA: n-oleoylethanolamine; PGC-1: PPAR γ co-activator 1; PKC: protein kinase C; POMC: pro-opiomelanocortin/cocaine- and amphetamine-regulated transcript; PPAR: peroxisomal proliferating activated receptor; RNS: reactive nitrogen species; ROS: reactive oxygen species; RXR: retinoid × receptor; SCAT: subcutaneous adipose tissue; SIRT 1: silent mating type information regulation 2 homologue 1; SNS: sympathetic nervous system; STAT-3: signal transducer and activator of transcription 3; SREBF1: sterol regulatory element-binding factor 1 gene; THC: tetrahydrocannabinol; TLR: toll-like receptor; TOR: target of rapamycin; TRPV1: transient receptor potential vanilloid type 1 receptor; T2D: type 2 diabetes; UCP-2: uncoupling protein 2; VAT: visceral adipose tissue; WAT: white adipose tissue.

## Competing interests

Alistair Nunn is a consultant to GW pharmaceuticals.

Jimmy Bell has no financial relationships to disclose.

Geoffrey Guy is the founder and chairman of GW pharmaceuticals.

## Authors' contributions

AN was the principle originator of the '*redox-thriftiness' *hypothesis and wrote and edited the manuscript. GG suggested the 'insulin resistance, friend or foe concept', which reinvigorated and focussed the final accepted version of the paper. GG acted as the senior author. JB and AN co-developed the tipping point concept, the LIMIT-AGE idea and suggested the possible function for VAT. GG and AN proposed the concept of evolutionary suicide. GG suggested the 'metabolic bowl' idea. JB emphasised the role of ectopic fat and metabolic inflexibility. AN proposed the polyphenol mode of action, the idea that polyunsaturated fat, alcohol, metformin and statins could have hormetic action, the possibility that inflammatory-driven prevention of weight gain could lead to accelerated ageing, and the possible mode of action of rimonabant. AN, JB and GG contributed equally to the further development and discussion of the all the hypotheses and all contributed to editorial suggestions. All authors read and approved the final manuscript.

## References

[B1] (2003). Diet, nutrition and the prevention of chronic diseases. World Health Organ Tech Rep Ser.

[B2] Zimmet P, Magliano D, Matsuzawa Y, Alberti G, Shaw J (2005). The metabolic syndrome: a global public health problem and a new definition. J Atheroscler Thromb.

[B3] Fujita K, Nishizawa H, Funahashi T, Shimomura I, Shimabukuro M (2006). Systemic oxidative stress is associated with visceral fat accumulation and the metabolic syndrome. Circ J.

[B4] Furukawa S, Fujita T, Shimabukuro M, Iwaki M, Yamada Y, Nakajima Y, Nakayama O, Makishima M, Matsuda M, Shimomura I (2004). Increased oxidative stress in obesity and its impact on metabolic syndrome. J Clin Invest.

[B5] Jeong SK, Kim YK, Park JW, Shin YJ, Kim DS (2008). Impact of visceral fat on the metabolic syndrome and nonalcoholic fatty liver disease. J Korean Med Sci.

[B6] van der PD, Milner KL, Hui J, Hodge A, Trenell MI, Kench JG, London R, Peduto T, Chisholm DJ, George J (2008). Visceral fat: a key mediator of steatohepatitis in metabolic liver disease. Hepatology.

[B7] Hotamisligil GS (2006). Inflammation and metabolic disorders. Nature.

[B8] Pasquali R, Vicennati V, Gambineri A, Pagotto U (2008). Sex-dependent role of glucocorticoids and androgens in the pathophysiology of human obesity. Int J Obes (Lond).

[B9] Storlien L, Oakes ND, Kelley DE (2004). Metabolic flexibility. Proc Nutr Soc.

[B10] Guarente L (2006). Sirtuins as potential targets for metabolic syndrome. Nature.

[B11] Murphy CT, McCarroll SA, Bargmann CI, Fraser A, Kamath RS, Ahringer J, Li H, Kenyon C (2003). Genes that act downstream of DAF-16 to influence the lifespan of Caenorhabditis elegans. Nature.

[B12] Nakae J, Biggs WH, Kitamura T, Cavenee WK, Wright CV, Arden KC, Accili D (2002). Regulation of insulin action and pancreatic beta-cell function by mutated alleles of the gene encoding forkhead transcription factor Foxo1. Nat Genet.

[B13] Dulloo AG (2006). Regulation of fat storage via suppressed thermogenesis: a thrifty phenotype that predisposes individuals with catch-up growth to insulin resistance and obesity. Horm Res.

[B14] Erol A (2007). Insulin resistance is an evolutionarily conserved physiological mechanism at the cellular level for protection against increased oxidative stress. Bioessays.

[B15] Fridlyand LE, Philipson LH (2006). Reactive species and early manifestation of insulin resistance in type 2 diabetes. Diabetes Obes Metab.

[B16] Stoger R (2008). The thrifty epigenotype: an acquired and heritable predisposition for obesity and diabetes?. Bioessays.

[B17] Sirikul B, Gower BA, Hunter GR, Larson-Meyer DE, Newcomer BR (2006). Relationship between insulin sensitivity and in vivo mitochondrial function in skeletal muscle. Am J Physiol Endocrinol Metab.

[B18] Wu CH, Heshka S, Wang J, Pierson RN, Heymsfield SB, Laferrere B, Wang Z, Albu JB, Pi-Sunyer X, Gallagher D (2007). Truncal fat in relation to total body fat: influences of age, sex, ethnicity and fatness. Int J Obes (Lond).

[B19] Fridlyand LE, Philipson LH (2006). Cold climate genes and the prevalence of type 2 diabetes mellitus. Med Hypotheses.

[B20] Civitarese AE, Carling S, Heilbronn LK, Hulver MH, Ukropcova B, Deutsch WA, Smith SR, Ravussin E (2007). Calorie restriction increases muscle mitochondrial biogenesis in healthy humans. PLoS Med.

[B21] Lopez-Lluch G, Hunt N, Jones B, Zhu M, Jamieson H, Hilmer S, Cascajo MV, Allard J, Ingram DK, Navas P, de CR (2006). Calorie restriction induces mitochondrial biogenesis and bioenergetic efficiency. Proc Natl Acad Sci USA.

[B22] Sreekumar R, Nair KS (2007). Skeletal muscle mitochondrial dysfunction & diabetes. Indian J Med Res.

[B23] Navarro A, Boveris A (2007). The mitochondrial energy transduction system and the aging process. Am J Physiol Cell Physiol.

[B24] NEEL JV (1962). Diabetes mellitus: a "thrifty" genotype rendered detrimental by "progress"?. Am J Hum Genet.

[B25] Reaven GM (1988). Banting lecture 1988. Role of insulin resistance in human disease. Diabetes.

[B26] Hales CN, Barker DJ (1992). Type 2 (non-insulin-dependent) diabetes mellitus: the thrifty phenotype hypothesis. Diabetologia.

[B27] Modi N, Thomas EL, Uthaya SN, Umranikar S, Bell JD, Yajnik C (2009). Whole body magnetic resonance imaging of healthy newborn infants demonstrates increased central adiposity in Asian Indians. Pediatr Res.

[B28] Prentice AM (2003). Intrauterine factors, adiposity, and hyperinsulinaemia. BMJ.

[B29] Bandyopadhyay GK, Yu JG, Ofrecio J, Olefsky JM (2005). Increased p85/55/50 expression and decreased phosphotidylinositol 3-kinase activity in insulin-resistant human skeletal muscle. Diabetes.

[B30] Takamura T, Honda M, Sakai Y, Ando H, Shimizu A, Ota T, Sakurai M, Misu H, Kurita S, Matsuzawa-Nagata N, Uchikata M, Nakamura S, Matoba R, Tanino M, Matsubara K, Kaneko S (2007). Gene expression profiles in peripheral blood mononuclear cells reflect the pathophysiology of type 2 diabetes. Biochem Biophys Res Commun.

[B31] Hotamisligil GS (2005). Role of endoplasmic reticulum stress and c-Jun NH2-terminal kinase pathways in inflammation and origin of obesity and diabetes. Diabetes.

[B32] Wang MC, Bohmann D, Jasper H (2005). JNK extends life span and limits growth by antagonizing cellular and organism-wide responses to insulin signaling. Cell.

[B33] Ni YG, Wang N, Cao DJ, Sachan N, Morris DJ, Gerard RD, Kuro O, Rothermel BA, Hill JA (2007). FoxO transcription factors activate Akt and attenuate insulin signaling in heart by inhibiting protein phosphatases. Proc Natl Acad Sci USA.

[B34] Oh SW, Mukhopadhyay A, Svrzikapa N, Jiang F, Davis RJ, Tissenbaum HA (2005). JNK regulates lifespan in Caenorhabditis elegans by modulating nuclear translocation of forkhead transcription factor/DAF-16. Proc Natl Acad Sci USA.

[B35] Huang H, Tindall DJ (2007). Dynamic FoxO transcription factors. J Cell Sci.

[B36] Morris BJ (2005). A forkhead in the road to longevity: the molecular basis of lifespan becomes clearer. J Hypertens.

[B37] Andersson U, Filipsson K, Abbott CR, Woods A, Smith K, Bloom SR, Carling D, Small CJ (2004). AMP-activated protein kinase plays a role in the control of food intake. J Biol Chem.

[B38] Kitamura T, Feng Y, Kitamura YI, Chua SC, Xu AW, Barsh GS, Rossetti L, Accili D (2006). Forkhead protein FoxO1 mediates Agrp-dependent effects of leptin on food intake. Nat Med.

[B39] Salih DA, Brunet A (2008). FoxO transcription factors in the maintenance of cellular homeostasis during aging. Curr Opin Cell Biol.

[B40] Corton JC, Brown-Borg HM (2005). Peroxisome proliferator-activated receptor gamma coactivator 1 in caloric restriction and other models of longevity. J Gerontol A Biol Sci Med Sci.

[B41] Corton JC, Apte U, Anderson SP, Limaye P, Yoon L, Latendresse J, Dunn C, Everitt JI, Voss KA, Swanson C, Kimbrough C, Wong JS, Gill SS, Chandraratna RA, Kwak MK, Kensler TW, Stulnig TM, Steffensen KR, Gustafsson JA, Mehendale HM (2004). Mimetics of caloric restriction include agonists of lipid-activated nuclear receptors. J Biol Chem.

[B42] St-Pierre J, Drori S, Uldry M, Silvaggi JM, Rhee J, Jager S, Handschin C, Zheng K, Lin J, Yang W, Simon DK, Bachoo R, Spiegelman BM (2006). Suppression of reactive oxygen species and neurodegeneration by the PGC-1 transcriptional coactivators. Cell.

[B43] Handschin C, Spiegelman BM (2008). The role of exercise and PGC1alpha in inflammation and chronic disease. Nature.

[B44] Vellai T (2009). Autophagy genes and ageing. Cell Death Differ.

[B45] Samuel VT, Choi CS, Phillips TG, Romanelli AJ, Geisler JG, Bhanot S, McKay R, Monia B, Shutter JR, Lindberg RA, Shulman GI, Veniant MM (2006). Targeting foxo1 in mice using antisense oligonucleotide improves hepatic and peripheral insulin action. Diabetes.

[B46] Gross DN, Heuvel AP van den, Birnbaum MJ (2008). The role of FoxO in the regulation of metabolism. Oncogene.

[B47] Nakae J, Cao Y, Oki M, Orba Y, Sawa H, Kiyonari H, Iskandar K, Suga K, Lombes M, Hayashi Y (2008). Forkhead transcription factor FoxO1 in adipose tissue regulates energy storage and expenditure. Diabetes.

[B48] Kitamura T, Nakae J, Kitamura Y, Kido Y, Biggs WH, Wright CV, White MF, Arden KC, Accili D (2002). The forkhead transcription factor Foxo1 links insulin signaling to Pdx1 regulation of pancreatic beta cell growth. J Clin Invest.

[B49] Woods SC, Benoit SC, Clegg DJ (2006). The Brain-Gut-Islet connection. Diabetes.

[B50] Lin X, Taguchi A, Park S, Kushner JA, Li F, Li Y, White MF (2004). Dysregulation of insulin receptor substrate 2 in beta cells and brain causes obesity and diabetes. J Clin Invest.

[B51] Carvalheira JB, Torsoni MA, Ueno M, Amaral ME, Araujo EP, Velloso LA, Gontijo JA, Saad MJ (2005). Cross-talk between the insulin and leptin signaling systems in rat hypothalamus. Obes Res.

[B52] Franks PW, Brage S, Luan J, Ekelund U, Rahman M, Farooqi IS, Halsall I, O'Rahilly S, Wareham NJ (2005). Leptin predicts a worsening of the features of the metabolic syndrome independently of obesity. Obes Res.

[B53] Erusalimsky JD, Moncada S (2007). Nitric oxide and mitochondrial signaling: from physiology to pathophysiology. Arterioscler Thromb Vasc Biol.

[B54] Linnane AW, Kios M, Vitetta L (2007). Healthy aging: regulation of the metabolome by cellular redox modulation and prooxidant signaling systems: the essential roles of superoxide anion and hydrogen peroxide. Biogerontology.

[B55] Ramsey MR, Sharpless NE (2006). ROS as a tumour suppressor?. Nat Cell Biol.

[B56] Essers MA, Weijzen S, de Vries-Smits AM, Saarloos I, de Ruiter ND, Bos JL, Burgering BM (2004). FOXO transcription factor activation by oxidative stress mediated by the small GTPase Ral and JNK. EMBO J.

[B57] Gomes AR, Brosens JJ, Lam EW (2008). Resist or die: FOXO transcription factors determine the cellular response to chemotherapy. Cell Cycle.

[B58] Nakamura T, Sakamoto K (2008). Forkhead transcription factor FOXO subfamily is essential for reactive oxygen species-induced apoptosis. Mol Cell Endocrinol.

[B59] Choi SL, Kim SJ, Lee KT, Kim J, Mu J, Birnbaum MJ, Soo KS, Ha J (2001). The regulation of AMP-activated protein kinase by H(2)O(2). Biochem Biophys Res Commun.

[B60] Sandstrom ME, Zhang SJ, Bruton J, Silva JP, Reid MB, Westerblad H, Katz A (2006). Role of reactive oxygen species in contraction-mediated glucose transport in mouse skeletal muscle. J Physiol.

[B61] Dandona P, Aljada A, Chaudhuri A, Mohanty P, Garg R (2005). Metabolic syndrome: a comprehensive perspective based on interactions between obesity, diabetes, and inflammation. Circulation.

[B62] Housley MP, Rodgers JT, Udeshi ND, Kelly TJ, Shabanowitz J, Hunt DF, Puigserver P, Hart GW (2008). O-GlcNAc regulates FoxO activation in response to glucose. J Biol Chem.

[B63] Hanover JA, Forsythe ME, Hennessey PT, Brodigan TM, Love DC, Ashwell G, Krause M (2005). A Caenorhabditis elegans model of insulin resistance: altered macronutrient storage and dauer formation in an OGT-1 knockout. Proc Natl Acad Sci USA.

[B64] Copeland RJ, Bullen JW, Hart GW (2008). Cross-talk between GlcNAcylation and phosphorylation: roles in insulin resistance and glucose toxicity. Am J Physiol Endocrinol Metab.

[B65] Weinkove D, Halstead JR, Gems D, Divecha N (2006). Long-term starvation and ageing induce AGE-1/PI 3-kinase-dependent translocation of DAF-16/FOXO to the cytoplasm. BMC Biol.

[B66] Blundell JE, Finlayson G (2004). Is susceptibility to weight gain characterized by homeostatic or hedonic risk factors for overconsumption?. Physiol Behav.

[B67] Jebb SA, Siervo M, Fruhbeck G, Goldberg GR, Murgatroyd PR, Prentice AM (2006). Variability of appetite control mechanisms in response to 9 weeks of progressive overfeeding in humans. Int J Obes (Lond).

[B68] Schwartz MW, Woods SC, Seeley RJ, Barsh GS, Baskin DG, Leibel RL (2003). Is the energy homeostasis system inherently biased toward weight gain?. Diabetes.

[B69] Alikhani M, Alikhani Z, Graves DT (2005). FOXO1 functions as a master switch that regulates gene expression necessary for tumor necrosis factor-induced fibroblast apoptosis. J Biol Chem.

[B70] Hu MC, Lee DF, Xia W, Golfman LS, Ou-Yang F, Yang JY, Zou Y, Bao S, Hanada N, Saso H, Kobayashi R, Hung MC (2004). IkappaB kinase promotes tumorigenesis through inhibition of forkhead FOXO3a. Cell.

[B71] De Souza CT, Araujo EP, Bordin S, Ashimine R, Zollner RL, Boschero AC, Saad MJ, Velloso LA (2005). Consumption of a fat-rich diet activates a proinflammatory response and induces insulin resistance in the hypothalamus. Endocrinology.

[B72] Zhang Y, Scarpace PJ (2006). The role of leptin in leptin resistance and obesity. Physiol Behav.

[B73] Andrews ZB, Liu ZW, Walllingford N, Erion DM, Borok E, Friedman JM, Tschop MH, Shanabrough M, Cline G, Shulman GI, Coppola A, Gao XB, Horvath TL, Diano S (2008). UCP2 mediates ghrelin's action on NPY/AgRP neurons by lowering free radicals. Nature.

[B74] Kola B, Farkas I, Christ-Crain M, Wittmann G, Lolli F, Amin F, Harvey-White J, Liposits Z, Kunos G, Grossman AB, Fekete C, Korbonits M (2008). The orexigenic effect of ghrelin is mediated through central activation of the endogenous cannabinoid system. PLoS ONE.

[B75] Volek JS, Feinman RD (2005). Carbohydrate restriction improves the features of Metabolic Syndrome. Metabolic Syndrome may be defined by the response to carbohydrate restriction. Nutr Metab (Lond).

[B76] Brownlee M (2001). Biochemistry and molecular cell biology of diabetic complications. Nature.

[B77] Maassen JA, Romijn JA, Heine RJ (2007). Fatty acid-induced mitochondrial uncoupling in adipocytes as a key protective factor against insulin resistance and beta cell dysfunction: a new concept in the pathogenesis of obesity-associated type 2 diabetes mellitus. Diabetologia.

[B78] Hudson NJ, Lehnert SA, Harper GS (2008). Obese humans as economically designed feed converters: symmorphosis and low oxidative capacity skeletal muscle. Med Hypotheses.

[B79] Tapia PC (2006). Sublethal mitochondrial stress with an attendant stoichiometric augmentation of reactive oxygen species may precipitate many of the beneficial alterations in cellular physiology produced by caloric restriction, intermittent fasting, exercise and dietary phytonutrients: "Mitohormesis" for health and vitality. Med Hypotheses.

[B80] Parsons PA (2007). Antagonistic pleiotropy and the stress theory of aging. Biogerontology.

[B81] Incerpi S, Fiore AM, De VP, Pedersen JZ (2007). Involvement of plasma membrane redox systems in hormone action. J Pharm Pharmacol.

[B82] Matsuzawa A, Ichijo H (2008). Redox control of cell fate by MAP kinase: physiological roles of ASK1-MAP kinase pathway in stress signaling. Biochim Biophys Acta.

[B83] Galli S, ntico Arciuch VG, Poderoso C, Converso DP, Zhou Q, Bal de Kier JE, Cadenas E, Boczkowski J, Carreras MC, Poderoso JJ (2008). Tumor cell phenotype is sustained by selective MAPK oxidation in mitochondria. PLoS ONE.

[B84] Brady NR, Elmore SP, van Beek JJ, Krab K, Courtoy PJ, Hue L, Westerhoff HV (2004). Coordinated behavior of mitochondria in both space and time: a reactive oxygen species-activated wave of mitochondrial depolarization. Biophys J.

[B85] Brookes PS, Yoon Y, Robotham JL, Anders MW, Sheu SS (2004). Calcium, ATP, and ROS: a mitochondrial love-hate triangle. Am J Physiol Cell Physiol.

[B86] Suliman HB, Carraway MS, Tatro LG, Piantadosi CA (2007). A new activating role for CO in cardiac mitochondrial biogenesis. J Cell Sci.

[B87] Menon SG, Goswami PC (2007). A redox cycle within the cell cycle: ring in the old with the new. Oncogene.

[B88] Brett PJ, Burtnick MN, Su H, Nair V, Gherardini FC (2008). iNOS activity is critical for the clearance of Burkholderia mallei from infected RAW 264.7 murine macrophages. Cell Microbiol.

[B89] Valerio A, Cardile A, Cozzi V, Bracale R, Tedesco L, Pisconti A, Palomba L, Cantoni O, Clementi E, Moncada S, Carruba MO, Nisoli E (2006). TNF-alpha downregulates eNOS expression and mitochondrial biogenesis in fat and muscle of obese rodents. J Clin Invest.

[B90] Cook S (2006). Coronary artery disease, nitric oxide and oxidative stress: the "Yin-Yang" effect–a Chinese concept for a worldwide pandemic. Swiss Med Wkly.

[B91] Nisoli E, Tonello C, Cardile A, Cozzi V, Bracale R, Tedesco L, Falcone S, Valerio A, Cantoni O, Clementi E, Moncada S, Carruba MO (2005). Calorie restriction promotes mitochondrial biogenesis by inducing the expression of eNOS. Science.

[B92] Hansen M, Chandra A, Mitic LL, Onken B, Driscoll M, Kenyon C (2008). A role for autophagy in the extension of lifespan by dietary restriction in C. elegans. PLoS Genet.

[B93] Jager S, Handschin C, St-Pierre J, Spiegelman BM (2007). AMP-activated protein kinase (AMPK) action in skeletal muscle via direct phosphorylation of PGC-1alpha. Proc Natl Acad Sci USA.

[B94] Rohas LM, St-Pierre J, Uldry M, Jager S, Handschin C, Spiegelman BM (2007). A fundamental system of cellular energy homeostasis regulated by PGC-1alpha. Proc Natl Acad Sci USA.

[B95] Cunningham JT, Rodgers JT, Arlow DH, Vazquez F, Mootha VK, Puigserver P (2007). mTOR controls mitochondrial oxidative function through a YY1-PGC-1alpha transcriptional complex. Nature.

[B96] Nemoto S, Fergusson MM, Finkel T (2005). SIRT1 functionally interacts with the metabolic regulator and transcriptional coactivator PGC-1{alpha}. J Biol Chem.

[B97] Frescas D, Valenti L, Accili D (2005). Nuclear trapping of the forkhead transcription factor FoxO1 via Sirt-dependent deacetylation promotes expression of glucogenetic genes. J Biol Chem.

[B98] Howitz KT, Bitterman KJ, Cohen HY, Lamming DW, Lavu S, Wood JG, Zipkin RE, Chung P, Kisielewski A, Zhang LL, Scherer B, Sinclair DA (2003). Small molecule activators of sirtuins extend Saccharomyces cerevisiae lifespan. Nature.

[B99] Rasbach KA, Schnellmann RG (2008). Isoflavones promote mitochondrial biogenesis. J Pharmacol Exp Ther.

[B100] Guarente L (2008). Mitochondria–a nexus for aging, calorie restriction, and sirtuins?. Cell.

[B101] Schulz TJ, Zarse K, Voigt A, Urban N, Birringer M, Ristow M (2007). Glucose restriction extends Caenorhabditis elegans life span by inducing mitochondrial respiration and increasing oxidative stress. Cell Metab.

[B102] Reznick RM, Shulman GI (2006). The role of AMP-activated protein kinase in mitochondrial biogenesis. J Physiol.

[B103] Olsen GS, Hansen BF (2002). AMP kinase activation ameliorates insulin resistance induced by free fatty acids in rat skeletal muscle. Am J Physiol Endocrinol Metab.

[B104] Kraegen EW, Saha AK, Preston E, Wilks D, Hoy AJ, Cooney GJ, Ruderman NB (2006). Increased malonyl-CoA and diacylglycerol content and reduced AMPK activity accompany insulin resistance induced by glucose infusion in muscle and liver of rats. Am J Physiol Endocrinol Metab.

[B105] Daval M, Foufelle F, Ferre P (2006). Functions of AMP-activated protein kinase in adipose tissue. J Physiol.

[B106] Steinberg GR (2007). Inflammation in obesity is the common link between defects in fatty acid metabolism and insulin resistance. Cell Cycle.

[B107] Greer EL, Oskoui PR, Banko MR, Maniar JM, Gygi MP, Gygi SP, Brunet A (2007). The energy sensor AMP-activated protein kinase directly regulates the mammalian FOXO3 transcription factor. J Biol Chem.

[B108] Fulco M, Sartorelli V (2008). Comparing and contrasting the roles of AMPK and SIRT1 in metabolic tissues. Cell Cycle.

[B109] Storozhevykh TP, Senilova YE, Persiyantseva NA, Pinelis VG, Pomytkin IA (2007). Mitochondrial respiratory chain is involved in insulin-stimulated hydrogen peroxide production and plays an integral role in insulin receptor autophosphorylation in neurons. BMC Neurosci.

[B110] Schieke SM, Phillips D, McCoy JP, Aponte AM, Shen RF, Balaban RS, Finkel T (2006). The mammalian target of rapamycin (mTOR) pathway regulates mitochondrial oxygen consumption and oxidative capacity. J Biol Chem.

[B111] Niedernhofer LJ, Robbins PD (2008). Signaling mechanisms involved in the response to genotoxic stress and regulating lifespan. Int J Biochem Cell Biol.

[B112] Dann SG, Selvaraj A, Thomas G (2007). mTOR Complex1-S6K1 signaling: at the crossroads of obesity, diabetes and cancer. Trends Mol Med.

[B113] Arany Z, Wagner BK, Ma Y, Chinsomboon J, Laznik D, Spiegelman BM (2008). Gene expression-based screening identifies microtubule inhibitors as inducers of PGC-1alpha and oxidative phosphorylation. Proc Natl Acad Sci USA.

[B114] Psarra AM, Sekeris CE (2008). Glucocorticoid receptors and other nuclear transcription factors in mitochondria and possible functions. Biochim Biophys Acta.

[B115] Weber K, Bruck P, Mikes Z, Kupper JH, Klingenspor M, Wiesner RJ (2002). Glucocorticoid hormone stimulates mitochondrial biogenesis specifically in skeletal muscle. Endocrinology.

[B116] Suliman HB, Welty-Wolf KE, Carraway M, Tatro L, Piantadosi CA (2004). Lipopolysaccharide induces oxidative cardiac mitochondrial damage and biogenesis. Cardiovasc Res.

[B117] Nunn AV, Bell J, Barter P (2007). The integration of lipid-sensing and anti-inflammatory effects: how the PPARs play a role in metabolic balance. Nucl Recept.

[B118] Adler AS, Sinha S, Kawahara TL, Zhang JY, Segal E, Chang HY (2007). Motif module map reveals enforcement of aging by continual NF-kappaB activity. Genes Dev.

[B119] Spencer NF, Poynter ME, Im SY, Daynes RA (1997). Constitutive activation of NF-kappa B in an animal model of aging. Int Immunol.

[B120] Bowles JT (1998). The evolution of aging: a new approach to an old problem of biology. Med Hypotheses.

[B121] Hadi HA, Carr CS, Al SJ (2005). Endothelial dysfunction: cardiovascular risk factors, therapy, and outcome. Vasc Health Risk Manag.

[B122] Lankin VZ, Tikhaze AK, Kapel'ko VI, Shepel'kova GS, Shumaev KB, Panasenko OM, Konovalova GG, Belenkov YN (2007). Mechanisms of oxidative modification of low density lipoproteins under conditions of oxidative and carbonyl stress. Biochemistry (Mosc).

[B123] Bhatt DL (2008). Anti-inflammatory agents and antioxidants as a possible "third great wave" in cardiovascular secondary prevention. Am J Cardiol.

[B124] Robert L, Robert AM, Fulop T (2008). Rapid increase in human life expectancy: will it soon be limited by the aging of elastin?. Biogerontology.

[B125] Dunbar JA, Reddy P, vis-Lameloise N, Philpot B, Laatikainen T, Kilkkinen A, Bunker SJ, Best JD, Vartiainen E, Kai LS, Janus ED (2008). Depression: an important comorbidity with metabolic syndrome in a general population. Diabetes Care.

[B126] Roriz-Filho S, Sa-Roriz TM, Rosset I, Camozzato AL, Santos AC, Chaves ML, Moriguti JC, Roriz-Cruz M (2008). (Pre)diabetes, brain aging, and cognition. Biochim Biophys Acta.

[B127] von MD, Safii S, Jassal SK, Svartberg J, Barrett-Connor E (2007). Associations between the metabolic syndrome and bone health in older men and women: the Rancho Bernardo Study. Osteoporos Int.

[B128] Wahba IM, Mak RH (2007). Obesity and obesity-initiated metabolic syndrome: mechanistic links to chronic kidney disease. Clin J Am Soc Nephrol.

[B129] Zhou JR, Blackburn GL, Walker WA (2007). Symposium introduction: metabolic syndrome and the onset of cancer. Am J Clin Nutr.

[B130] Fernandez-Real JM, Ricart W (1999). Insulin resistance and inflammation in an evolutionary perspective: the contribution of cytokine genotype/phenotype to thriftiness. Diabetologia.

[B131] Demas GE, Sakaria S (2005). Leptin regulates energetic tradeoffs between body fat and humoural immunity. Proc Biol Sci.

[B132] Duncan BB, Schmidt MI (2001). Chronic activation of the innate immune system may underlie the metabolic syndrome. Sao Paulo Med J.

[B133] Pickup JC, Mattock MB, Chusney GD, Burt D (1997). NIDDM as a disease of the innate immune system: association of acute-phase reactants and interleukin-6 with metabolic syndrome X. Diabetologia.

[B134] Bjorntorp P, Rosmond R (1999). Visceral obesity and diabetes. Drugs.

[B135] Evans JL, Goldfine ID, Maddux BA, Grodsky GM (2002). Oxidative stress and stress-activated signaling pathways: a unifying hypothesis of type 2 diabetes. Endocr Rev.

[B136] Solinas G, Vilcu C, Neels JG, Bandyopadhyay GK, Luo JL, Naugler W, Grivennikov S, Wynshaw-Boris A, Scadeng M, Olefsky JM, Karin M (2007). JNK1 in hematopoietically derived cells contributes to diet-induced inflammation and insulin resistance without affecting obesity. Cell Metab.

[B137] Tuncman G, Hirosumi J, Solinas G, Chang L, Karin M, Hotamisligil GS (2006). Functional in vivo interactions between JNK1 and JNK2 isoforms in obesity and insulin resistance. Proc Natl Acad Sci USA.

[B138] Hirosumi J, Tuncman G, Chang L, Gorgun CZ, Uysal KT, Maeda K, Karin M, Hotamisligil GS (2002). A central role for JNK in obesity and insulin resistance. Nature.

[B139] Bubici C, Papa S, Dean K, Franzoso G (2006). Mutual cross-talk between reactive oxygen species and nuclear factor-kappa B: molecular basis and biological significance. Oncogene.

[B140] Nakano H, Nakajima A, Sakon-Komazawa S, Piao JH, Xue X, Okumura K (2006). Reactive oxygen species mediate crosstalk between NF-kappaB and JNK. Cell Death Differ.

[B141] Cinti S, Mitchell G, Barbatelli G, Murano I, Ceresi E, Faloia E, Wang S, Fortier M, Greenberg AS, Obin MS (2005). Adipocyte death defines macrophage localization and function in adipose tissue of obese mice and humans. J Lipid Res.

[B142] Spalding KL, Arner E, Westermark PO, Bernard S, Buchholz BA, Bergmann O, Blomqvist L, Hoffstedt J, Naslund E, Britton T, Concha H, Hassan M, Ryden M, Frisen J, Arner P (2008). Dynamics of fat cell turnover in humans. Nature.

[B143] Kim JY, van de WE, Laplante M, Azzara A, Trujillo ME, Hofmann SM, Schraw T, Durand JL, Li H, Li G, Jelicks LA, Mehler MF, Hui DY, Deshaies Y, Shulman GI, Schwartz GJ, Scherer PE (2007). Obesity-associated improvements in metabolic profile through expansion of adipose tissue. J Clin Invest.

[B144] Dowell P, Otto TC, Adi S, Lane MD (2003). Convergence of peroxisome proliferator-activated receptor gamma and Foxo1 signaling pathways. J Biol Chem.

[B145] Delerive P, De BK, Besnard S, Vanden BW, Peters JM, Gonzalez FJ, Fruchart JC, Tedgui A, Haegeman G, Staels B (1999). Peroxisome proliferator-activated receptor alpha negatively regulates the vascular inflammatory gene response by negative cross-talk with transcription factors NF-kappaB and AP-1. J Biol Chem.

[B146] Planavila A, Rodriguez-Calvo R, Jove M, Michalik L, Wahli W, Laguna JC, Vazquez-Carrera M (2005). Peroxisome proliferator-activated receptor beta/delta activation inhibits hypertrophy in neonatal rat cardiomyocytes. Cardiovasc Res.

[B147] Rival Y, Beneteau N, Taillandier T, Pezet M, Dupont-Passelaigue E, Patoiseau JF, Junquero D, Colpaert FC, Delhon A (2002). PPARalpha and PPARdelta activators inhibit cytokine-induced nuclear translocation of NF-kappaB and expression of VCAM-1 in EAhy926 endothelial cells. Eur J Pharmacol.

[B148] Tham DM, Martin-McNulty B, Wang YX, Wilson DW, Vergona R, Sullivan ME, Dole W, Rutledge JC (2002). Angiotensin II is associated with activation of NF-kappaB-mediated genes and downregulation of PPARs. Physiol Genomics.

[B149] Kim JE, Chen J (2004). regulation of peroxisome proliferator-activated receptor-gamma activity by mammalian target of rapamycin and amino acids in adipogenesis. Diabetes.

[B150] Laferrere B, Abraham C, Awad M, Jean-Baptiste S, Hart AB, Garcia-Lorda P, Kokkoris P, Russell CD (2006). Inhibiting endogenous cortisol blunts the meal-entrained rise in serum leptin. J Clin Endocrinol Metab.

[B151] Eikelis N, Esler M (2005). The neurobiology of human obesity. Exp Physiol.

[B152] Fehm HL, Kern W, Peters A (2006). The selfish brain: competition for energy resources. Prog Brain Res.

[B153] Haussmann MF, Vleck CM, Farrar ES (2007). A laboratory exercise to illustrate increased salivary cortisol in response to three stressful conditions using competitive ELISA. Adv Physiol Educ.

[B154] Malcher-Lopes R, Franco A, Tasker JG (2008). Glucocorticoids shift arachidonic acid metabolism toward endocannabinoid synthesis: a non-genomic anti-inflammatory switch. Eur J Pharmacol.

[B155] Esteve E, Ricart W, Fernandez-Real JM (2005). Dyslipidemia and inflammation: an evolutionary conserved mechanism. Clin Nutr.

[B156] Khovidhunkit W, Kim MS, Memon RA, Shigenaga JK, Moser AH, Feingold KR, Grunfeld C (2004). Effects of infection and inflammation on lipid and lipoprotein metabolism: mechanisms and consequences to the host. J Lipid Res.

[B157] Packard C, Nunn A, Hobbs R (2002). High density lipoprotein: guardian of the vascular system?. Int J Clin Pract.

[B158] Wajchenberg BL (2000). Subcutaneous and visceral adipose tissue: their relation to the metabolic syndrome. Endocr Rev.

[B159] Navab M, Ananthramaiah GM, Reddy ST, Van Lenten BJ, Ansell BJ, Fonarow GC, Vahabzadeh K, Hama S, Hough G, Kamranpour N, Berliner JA, Lusis AJ, Fogelman AM (2004). The oxidation hypothesis of atherogenesis: the role of oxidized phospholipids and HDL. J Lipid Res.

[B160] Eizirik DL, Cardozo AK, Cnop M (2008). The role for endoplasmic reticulum stress in diabetes mellitus. Endocr Rev.

[B161] Zhang K, Kaufman RJ (2008). From endoplasmic-reticulum stress to the inflammatory response. Nature.

[B162] Muzumdar R, Allison DB, Huffman DM, Ma X, Atzmon G, Einstein FH, Fishman S, Poduval AD, McVei T, Keith SW, Barzilai N (2008). Visceral adipose tissue modulates mammalian longevity. Aging Cell.

[B163] Freedland ES (2004). Role of a critical visceral adipose tissue threshold (CVATT) in metabolic syndrome: implications for controlling dietary carbohydrates: a review. Nutr Metab (Lond).

[B164] Varady KA, Hellerstein MK (2007). Alternate-day fasting and chronic disease prevention: a review of human and animal trials. Am J Clin Nutr.

[B165] Allard JS, Heilbronn LK, Smith C, Hunt ND, Ingram DK, Ravussin E, de CR (2008). In vitro cellular adaptations of indicators of longevity in response to treatment with serum collected from humans on calorie restricted diets. PLoS ONE.

[B166] Varady KA, Roohk DJ, Loe YC, Evoy-Hein BK, Hellerstein MK (2007). Effects of modified alternate-day fasting regimens on adipocyte size, triglyceride metabolism, and plasma adiponectin levels in mice. J Lipid Res.

[B167] Wang CS, Wang ST, Yao WJ, Chang TT, Chou P (2007). Hepatitis C virus infection and the development of type 2 diabetes in a community-based longitudinal study. Am J Epidemiol.

[B168] Hu FB (2003). Plant-based foods and prevention of cardiovascular disease: an overview. Am J Clin Nutr.

[B169] Simon JA, Hodgkins ML, Browner WS, Neuhaus JM, Bernert JT, Hulley SB (1995). Serum fatty acids and the risk of coronary heart disease. Am J Epidemiol.

[B170] Skerrett PJ, Hennekens CH (2003). Consumption of fish and fish oils and decreased risk of stroke. Prev Cardiol.

[B171] Eaton SB (1992). Humans, lipids and evolution. Lipids.

[B172] Eaton SB, Eaton SB, Sinclair AJ, Cordain L, Mann NJ (1998). Dietary intake of long-chain polyunsaturated fatty acids during the paleolithic. World Rev Nutr Diet.

[B173] Heywood PF (1977). The public health significance of fat-modified ruminant foods. Am J Clin Nutr.

[B174] Pietinen P, Uusitalo U, Vartiainen E, Tuomilehto J (1988). Dietary survey of the FINMONICA project in 1982. Acta Med Scand Suppl.

[B175] Robinson I, de Serna DG, Gutierrez A, Schade DS (2006). Vitamin E in humans: an explanation of clinical trial failure. Endocr Pract.

[B176] Cosgrove JP, Church DF, Pryor WA (1987). The kinetics of the autoxidation of polyunsaturated fatty acids. Lipids.

[B177] Tsalouhidou S, Argyrou C, Theofilidis G, Karaoglanidis D, Orfanidou E, Nikolaidis MG, Petridou A, Mougios V (2006). Mitochondrial phospholipids of rat skeletal muscle are less polyunsaturated than whole tissue phospholipids: implications for protection against oxidative stress. J Anim Sci.

[B178] Esterbauer H, Schaur RJ, Zollner H (1991). Chemistry and biochemistry of 4-hydroxynonenal, malonaldehyde and related aldehydes. Free Radic Biol Med.

[B179] Ghosh S, Kewalramani G, Yuen G, Pulinilkunnil T, An D, Innis SM, Allard MF, Wambolt RB, Qi D, Abrahani A, Rodrigues B (2006). Induction of mitochondrial nitrative damage and cardiac dysfunction by chronic provision of dietary omega-6 polyunsaturated fatty acids. Free Radic Biol Med.

[B180] Zhang BX, Ma X, Zhang W, Yeh CK, Lin A, Luo J, Sprague EA, Swerdlow RH, Katz MS (2006). Polyunsaturated fatty acids mobilize intracellular Ca2+ in NT2 human teratocarcinoma cells by causing release of Ca2+ from mitochondria. Am J Physiol Cell Physiol.

[B181] Ochoa JJ, Quiles JL, Lopez-Frias M, Huertas JR, Mataix J (2007). Effect of lifelong coenzyme Q10 supplementation on age-related oxidative stress and mitochondrial function in liver and skeletal muscle of rats fed on a polyunsaturated fatty acid (PUFA)-rich diet. J Gerontol A Biol Sci Med Sci.

[B182] Hilakivi-Clarke L, Olivo SE, Shajahan A, Khan G, Zhu Y, Zwart A, Cho E, Clarke R (2005). Mechanisms mediating the effects of prepubertal (n-3) polyunsaturated fatty acid diet on breast cancer risk in rats. J Nutr.

[B183] Xu HE, Lambert MH, Montana VG, Parks DJ, Blanchard SG, Brown PJ, Sternbach DD, Lehmann JM, Wisely GB, Willson TM, Kliewer SA, Milburn MV (1999). Molecular recognition of fatty acids by peroxisome proliferator-activated receptors. Mol Cell.

[B184] Reilly JM, Thompson MP (2000). Dietary fatty acids Up-regulate the expression of UCP2 in 3T3-L1 preadipocytes. Biochem Biophys Res Commun.

[B185] Armstrong MB, Towle HC (2001). Polyunsaturated fatty acids stimulate hepatic UCP-2 expression via a PPARalpha-mediated pathway. Am J Physiol Endocrinol Metab.

[B186] Echtay KS (2007). Mitochondrial uncoupling proteins–what is their physiological role?. Free Radic Biol Med.

[B187] Weigert C, Brodbeck K, Staiger H, Kausch C, Machicao F, Haring HU, Schleicher ED (2004). Palmitate, but not unsaturated fatty acids, induces the expression of interleukin-6 in human myotubes through proteasome-dependent activation of nuclear factor-kappaB. J Biol Chem.

[B188] Han DH, Nolte LA, Ju JS, Coleman T, Holloszy JO, Semenkovich CF (2004). UCP-mediated energy depletion in skeletal muscle increases glucose transport despite lipid accumulation and mitochondrial dysfunction. Am J Physiol Endocrinol Metab.

[B189] Bastie CC, Hajri T, Drover VA, Grimaldi PA, Abumrad NA (2004). CD36 in myocytes channels fatty acids to a lipase-accessible triglyceride pool that is related to cell lipid and insulin responsiveness. Diabetes.

[B190] Schrauwen P, Hesselink MK (2004). Oxidative capacity, lipotoxicity, and mitochondrial damage in type 2 diabetes. Diabetes.

[B191] Schrauwen P, Hesselink M (2002). UCP2 and UCP3 in muscle controlling body metabolism. J Exp Biol.

[B192] Mootha VK, Lindgren CM, Eriksson KF, Subramanian A, Sihag S, Lehar J, Puigserver P, Carlsson E, Ridderstrale M, Laurila E, Houstis N, Daly MJ, Patterson N, Mesirov JP, Golub TR, Tamayo P, Spiegelman B, Lander ES, Hirschhorn JN, Altshuler D, Groop LC (2003). PGC-1alpha-responsive genes involved in oxidative phosphorylation are coordinately downregulated in human diabetes. Nat Genet.

[B193] Carroll AM, Porter RK (2004). Starvation-sensitive UCP 3 protein expression in thymus and spleen mitochondria. Biochim Biophys Acta.

[B194] Sullivan PG, Rippy NA, Dorenbos K, Concepcion RC, Agarwal AK, Rho JM (2004). The ketogenic diet increases mitochondrial uncoupling protein levels and activity. Ann Neurol.

[B195] Xiao H, Massaro D, Massaro GD, Clerch LB (2004). Expression of lung uncoupling protein-2 mRNA is modulated developmentally and by caloric intake. Exp Biol Med (Maywood).

[B196] Stein DT, Stevenson BE, Chester MW, Basit M, Daniels MB, Turley SD, McGarry JD (1997). The insulinotropic potency of fatty acids is influenced profoundly by their chain length and degree of saturation. J Clin Invest.

[B197] Ito E, Ozawa S, Takahashi K, Tanaka T, Katsuta H, Yamaguchi S, Maruyama M, Takizawa M, Katahira H, Yoshimoto K, Nagamatsu S, Ishida H (2004). PPAR-gamma overexpression selectively suppresses insulin secretory capacity in isolated pancreatic islets through induction of UCP-2 protein. Biochem Biophys Res Commun.

[B198] Gremlich S, Nolan C, Roduit R, Burcelin R, Peyot ML, ghingaro-Augusto V, Desvergne B, Michalik L, Prentki M, Wahli W (2005). Pancreatic islet adaptation to fasting is dependent on peroxisome proliferator-activated receptor alpha transcriptional up-regulation of fatty acid oxidation. Endocrinology.

[B199] Lee CH, Olson P, Hevener A, Mehl I, Chong LW, Olefsky JM, Gonzalez FJ, Ham J, Kang H, Peters JM, Evans RM (2006). PPARdelta regulates glucose metabolism and insulin sensitivity. Proc Natl Acad Sci USA.

[B200] Haluzik MM, Lacinova Z, Dolinkova M, Haluzikova D, Housa D, Horinek A, Vernerova Z, Kumstyrova T, Haluzik M (2006). Improvement of Insulin Sensitivity after Peroxisome Proliferator-Activated Receptor-{alpha} Agonist Treatment Is Accompanied by Paradoxical Increase of Circulating Resistin Levels. Endocrinology.

[B201] Ye JM, Doyle PJ, Iglesias MA, Watson DG, Cooney GJ, Kraegen EW (2001). Peroxisome proliferator-activated receptor (PPAR)-alpha activation lowers muscle lipids and improves insulin sensitivity in high fat-fed rats: comparison with PPAR-gamma activation. Diabetes.

[B202] Van LF, Liang X, Andris F, Urbain J, Vandenbranden M, Ruysschaert JM, Resh MD, Stulnig TM, Leo O (2003). Glucocorticoids alter the lipid and protein composition of membrane rafts of a murine T cell hybridoma. J Immunol.

[B203] Lee JY, Hwang DH (2006). The modulation of inflammatory gene expression by lipids: mediation through Toll-like receptors. Mol Cells.

[B204] Shi H, Kokoeva MV, Inouye K, Tzameli I, Yin H, Flier JS (2006). TLR4 links innate immunity and fatty acid-induced insulin resistance. J Clin Invest.

[B205] Ma DW (2007). Lipid mediators in membrane rafts are important determinants of human health and disease. Appl Physiol Nutr Metab.

[B206] Mathias S, Pena LA, Kolesnick RN (1998). Signal transduction of stress via ceramide. Biochem J.

[B207] Schmitz-Peiffer C, Craig DL, Biden TJ (1999). Ceramide generation is sufficient to account for the inhibition of the insulin-stimulated PKB pathway in C2C12 skeletal muscle cells pretreated with palmitate. J Biol Chem.

[B208] Lin J, Yang R, Tarr PT, Wu PH, Handschin C, Li S, Yang W, Pei L, Uldry M, Tontonoz P, Newgard CB, Spiegelman BM (2005). Hyperlipidemic effects of dietary saturated fats mediated through PGC-1beta coactivation of SREBP. Cell.

[B209] Hirasawa A, Tsumaya K, Awaji T, Katsuma S, Adachi T, Yamada M, Sugimoto Y, Miyazaki S, Tsujimoto G (2005). Free fatty acids regulate gut incretin glucagon-like peptide-1 secretion through GPR120. Nat Med.

[B210] Haag M, Dippenaar NG (2005). Dietary fats, fatty acids and insulin resistance: short review of a multifaceted connection. Med Sci Monit.

[B211] Wang H, Storlien LH, Huang XF (2002). Effects of dietary fat types on body fatness, leptin, and ARC leptin receptor, NPY, and AgRP mRNA expression. Am J Physiol Endocrinol Metab.

[B212] Rivellese AA, Lilli S (2003). Quality of dietary fatty acids, insulin sensitivity and type 2 diabetes. Biomed Pharmacother.

[B213] Marcheselli VL, Hong S, Lukiw WJ, Tian XH, Gronert K, Musto A, Hardy M, Gimenez JM, Chiang N, Serhan CN, Bazan NG (2003). Novel docosanoids inhibit brain ischemia-reperfusion-mediated leukocyte infiltration and pro-inflammatory gene expression. J Biol Chem.

[B214] Mechoulam R, Spatz M, Shohami E (2002). Endocannabinoids and neuroprotection. Sci STKE.

[B215] Yin W, Signore AP, Iwai M, Cao G, Gao Y, Chen J (2008). Rapidly increased neuronal mitochondrial biogenesis after hypoxic-ischemic brain injury. Stroke.

[B216] Viveros MP, de Fonseca FR, Bermudez-Silva FJ, McPartland JM (2008). Critical role of the endocannabinoid system in the regulation of food intake and energy metabolism, with phylogenetic, developmental, and pathophysiological implications. Endocr Metab Immune Disord Drug Targets.

[B217] Di MV, Matias I (2005). Endocannabinoid control of food intake and energy balance. Nat Neurosci.

[B218] Di MV, Szallasi A (2008). Rimonabant in rats with a metabolic syndrome: good news after the depression. Br J Pharmacol.

[B219] Doyon C, Denis RG, Baraboi ED, Samson P, Lalonde J, Deshaies Y, Richard D (2006). Effects of rimonabant (SR141716) on fasting-induced hypothalamic-pituitary-adrenal axis and neuronal activation in lean and obese Zucker rats. Diabetes.

[B220] Steiner MA, Marsicano G, Nestler EJ, Holsboer F, Lutz B, Wotjak CT (2008). Antidepressant-like behavioral effects of impaired cannabinoid receptor type 1 signaling coincide with exaggerated corticosterone secretion in mice. Psychoneuroendocrinology.

[B221] Motaghedi R, McGraw TE (2008). The CB1 endocannabinoid system modulates adipocyte insulin sensitivity. Obesity (Silver Spring).

[B222] Eckardt K, Sell H, Taube A, Koenen M, Platzbecker B, Cramer A, Horrighs A, Lehtonen M, Tennagels N, Eckel J (2009). Cannabinoid type 1 receptors in human skeletal muscle cells participate in the negative crosstalk between fat and muscle. Diabetologia.

[B223] Jbilo O, Ravinet-Trillou C, Arnone M, Buisson I, Bribes E, Peleraux A, Penarier G, Soubrie P, Le FG, Galiegue S, Casellas P (2005). The CB1 receptor antagonist rimonabant reverses the diet-induced obesity phenotype through the regulation of lipolysis and energy balance. FASEB J.

[B224] Tedesco L, Valerio A, Cervino C, Cardile A, Pagano C, Vettor R, Pasquali R, Carruba MO, Marsicano G, Lutz B, Pagotto U, Nisoli E (2008). Cannabinoid type 1 receptor blockade promotes mitochondrial biogenesis through endothelial nitric oxide synthase expression in white adipocytes. Diabetes.

[B225] Curioni C, Andre C (2006). Rimonabant for overweight or obesity. Cochrane Database Syst Rev.

[B226] Zimmer A, Zimmer AM, Hohmann AG, Herkenham M, Bonner TI (1999). Increased mortality, hypoactivity, and hypoalgesia in cannabinoid CB1 receptor knockout mice. Proc Natl Acad Sci USA.

[B227] Kola B, Hubina E, Tucci SA, Kirkham TC, Garcia EA, Mitchell SE, Williams LM, Hawley SA, Hardie DG, Grossman AB, Korbonits M (2005). Cannabinoids and ghrelin have both central and peripheral metabolic and cardiac effects via AMP-activated protein kinase. J Biol Chem.

[B228] O'Sullivan SE (2007). Cannabinoids go nuclear: evidence for activation of peroxisome proliferator-activated receptors. Br J Pharmacol.

[B229] Du X, Matsumura T, Edelstein D, Rossetti L, Zsengeller Z, Szabo C, Brownlee M (2003). Inhibition of GAPDH activity by poly(ADP-ribose) polymerase activates three major pathways of hyperglycemic damage in endothelial cells. J Clin Invest.

[B230] Lavrovsky Y, Chatterjee B, Clark RA, Roy AK (2000). Role of redox-regulated transcription factors in inflammation, aging and age-related diseases. Exp Gerontol.

[B231] Yeh CH, Sturgis L, Haidacher J, Zhang XN, Sherwood SJ, Bjercke RJ, Juhasz O, Crow MT, Tilton RG, Denner L (2001). Requirement for p38 and p44/p42 mitogen-activated protein kinases in RAGE-mediated nuclear factor-kappaB transcriptional activation and cytokine secretion. Diabetes.

[B232] Lin Y, Rajala MW, Berger JP, Moller DE, Barzilai N, Scherer PE (2001). Hyperglycemia-induced production of acute phase reactants in adipose tissue. J Biol Chem.

[B233] Dandona P, Aljada A, Dhindsa S, Garg R (2003). Insulin as an anti-inflammatory and antiatherosclerotic hormone. Clin Cornerstone.

[B234] Accurso A, Bernstein RK, Dahlqvist A, Draznin B, Feinman RD, Fine EJ, Gleed A, Jacobs DB, Larson G, Lustig RH, Manninen AH, McFarlane SI, Morrison K, Nielsen JV, Ravnskov U, Roth KS, Silvestre R, Sowers JR, Sundberg R, Volek JS, Westman EC, Wood RJ, Wortman J, Vernon MC (2008). Dietary carbohydrate restriction in type 2 diabetes mellitus and metabolic syndrome: time for a critical appraisal. Nutr Metab (Lond).

[B235] Shay NF, Banz WJ (2005). Regulation of gene transcription by botanicals: novel regulatory mechanisms. Annu Rev Nutr.

[B236] Lin YL, Lin JK (1997). (-)-Epigallocatechin-3-gallate blocks the induction of nitric oxide synthase by down-regulating lipopolysaccharide-induced activity of transcription factor nuclear factor-kappaB. Mol Pharmacol.

[B237] Anton S, Melville L, Rena G (2007). Epigallocatechin gallate (EGCG) mimics insulin action on the transcription factor FOXO1a and elicits cellular responses in the presence and absence of insulin. Cell Signal.

[B238] Wood JG, Rogina B, Lavu S, Howitz K, Helfand SL, Tatar M, Sinclair D (2004). Sirtuin activators mimic caloric restriction and delay ageing in metazoans. Nature.

[B239] Mercader J, Ribot J, Murano I, Felipe F, Cinti S, Bonet ML, Palou A (2006). Remodeling of white adipose tissue after retinoic acid administration in mice. Endocrinology.

[B240] Zomer AW, van Der BB, Jansen GA, Wanders RJ, Poll-The BT, Saag PT van Der (2000). Pristanic acid and phytanic acid: naturally occurring ligands for the nuclear receptor peroxisome proliferator-activated receptor alpha. J Lipid Res.

[B241] Szanto A, Narkar V, Shen Q, Uray IP, Davies PJ, Nagy L (2004). Retinoid × receptors: X-ploring their (patho)physiological functions. Cell Death Differ.

[B242] Austenaa LM, Carlsen H, Ertesvag A, Alexander G, Blomhoff HK, Blomhoff R (2004). Vitamin A status significantly alters nuclear factor-kappaB activity assessed by in vivo imaging. FASEB J.

[B243] Yoon JH, Baek SJ (2005). Molecular targets of dietary polyphenols with anti-inflammatory properties. Yonsei Med J.

[B244] Shao HB, Chu LY, Shao MA, Jaleel CA, Mi HM (2008). Higher plant antioxidants and redox signaling under environmental stresses. C R Biol.

[B245] Jimenez C, Cossio BR, Rivard CJ, Berl T, Capasso JM (2007). Cell division in the unicellular microalga Dunaliella viridis depends on phosphorylation of extracellular signal-regulated kinases (ERKs). J Exp Bot.

[B246] Briviba K, Pan L, Rechkemmer G (2002). Red wine polyphenols inhibit the growth of colon carcinoma cells and modulate the activation pattern of mitogen-activated protein kinases. J Nutr.

[B247] Oak MH, Bedoui JE, Madeira SV, Chalupsky K, Schini-Kerth VB (2006). Delphinidin and cyanidin inhibit PDGF(AB)-induced VEGF release in vascular smooth muscle cells by preventing activation of p38 MAPK and JNK. Br J Pharmacol.

[B248] Zang M, Xu S, Maitland-Toolan KA, Zuccollo A, Hou X, Jiang B, Wierzbicki M, Verbeuren TJ, Cohen RA (2006). Polyphenols stimulate AMP-activated protein kinase, lower lipids, and inhibit accelerated atherosclerosis in diabetic LDL receptor-deficient mice. Diabetes.

[B249] Zheng J, Ramirez VD (2000). Inhibition of mitochondrial proton F0F1-ATPase/ATP synthase by polyphenolic phytochemicals. Br J Pharmacol.

[B250] Athanasiou A, Clarke AB, Turner AE, Kumaran NM, Vakilpour S, Smith PA, Bagiokou D, Bradshaw TD, Westwell AD, Fang L, Lobo DN, Constantinescu CS, Calabrese V, Loesch A, Alexander SP, Clothier RH, Kendall DA, Bates TE (2007). Cannabinoid receptor agonists are mitochondrial inhibitors: a unified hypothesis of how cannabinoids modulate mitochondrial function and induce cell death. Biochem Biophys Res Commun.

[B251] Klinge CM, Blankenship KA, Risinger KE, Bhatnagar S, Noisin EL, Sumanasekera WK, Zhao L, Brey DM, Keynton RS (2005). Resveratrol and estradiol rapidly activate MAPK signaling through estrogen receptors alpha and beta in endothelial cells. J Biol Chem.

[B252] Wallerath T, Deckert G, Ternes T, Anderson H, Li H, Witte K, Forstermann U (2002). Resveratrol, a polyphenolic phytoalexin present in red wine, enhances expression and activity of endothelial nitric oxide synthase. Circulation.

[B253] Razmara A, Sunday L, Stirone C, Wang X, Krause D, Duckles S, Procaccio V (2008). Mitochondrial effects of estrogen are mediated by ER{alpha} in brain endothelial cells. J Pharmacol Exp Ther.

[B254] Kutuk O, Poli G, Basaga H (2006). Resveratrol protects against 4-hydroxynonenal-induced apoptosis by blocking JNK and c-JUN/AP-1 signaling. Toxicol Sci.

[B255] Zhang J (2006). Resveratrol inhibits insulin responses in a SirT1-independent pathway. Biochem J.

[B256] Baur JA, Pearson KJ, Price NL, Jamieson HA, Lerin C, Kalra A, Prabhu VV, Allard JS, Lopez-Lluch G, Lewis K, Pistell PJ, Poosala S, Becker KG, Boss O, Gwinn D, Wang M, Ramaswamy S, Fishbein KW, Spencer RG, Lakatta EG, Le CD, Shaw RJ, Navas P, Puigserver P, Ingram DK, de CR, Sinclair DA (2006). Resveratrol improves health and survival of mice on a high-calorie diet. Nature.

[B257] Valenzano DR, Terzibasi E, Genade T, Cattaneo A, Domenici L, Cellerino A (2006). Resveratrol prolongs lifespan and retards the onset of age-related markers in a short-lived vertebrate. Curr Biol.

[B258] Lamming DW, Wood JG, Sinclair DA (2004). Small molecules that regulate lifespan: evidence for xenohormesis. Mol Microbiol.

[B259] Narkar VA, Downes M, Yu RT, Embler E, Wang YX, Banayo E, Mihaylova MM, Nelson MC, Zou Y, Juguilon H, Kang H, Shaw RJ, Evans RM (2008). AMPK and PPARdelta agonists are exercise mimetics. Cell.

[B260] Lagouge M, Argmann C, Gerhart-Hines Z, Meziane H, Lerin C, Daussin F, Messadeq N, Milne J, Lambert P, Elliott P, Geny B, Laakso M, Puigserver P, Auwerx J (2006). Resveratrol improves mitochondrial function and protects against metabolic disease by activating SIRT1 and PGC-1alpha. Cell.

[B261] Golay A (2008). Metformin and body weight. Int J Obes (Lond).

[B262] Zou MH, Kirkpatrick SS, Davis BJ, Nelson JS, Wiles WG, Schlattner U, Neumann D, Brownlee M, Freeman MB, Goldman MH (2004). Activation of the AMP-activated protein kinase by the anti-diabetic drug metformin in vivo. Role of mitochondrial reactive nitrogen species. J Biol Chem.

[B263] Suwa M, Egashira T, Nakano H, Sasaki H, Kumagai S (2006). Metformin increases the PGC-1alpha protein and oxidative enzyme activities possibly via AMPK phosphorylation in skeletal muscle in vivo. J Appl Physiol.

[B264] Singh U, Devaraj S, Jialal I, Siegel D (2008). Comparison effect of atorvastatin (10 versus 80 mg) on biomarkers of inflammation and oxidative stress in subjects with metabolic syndrome. Am J Cardiol.

[B265] Strazzullo P, Kerry SM, Barbato A, Versiero M, D'Elia L, Cappuccio FP (2007). Do statins reduce blood pressure?: a meta-analysis of randomized, controlled trials. Hypertension.

[B266] Guclu F, Ozmen B, Hekimsoy Z, Kirmaz C (2004). Effects of a statin group drug, pravastatin, on the insulin resistance in patients with metabolic syndrome. Biomed Pharmacother.

[B267] Sasaki J, Ikeda Y, Kuribayashi T, Kajiwara K, Biro S, Yamamoto K, Ageta M, Kobori S, Saikawa T, Otonari T, Kono S (2008). A 52-week, randomized, open-label, parallel-group comparison of the tolerability and effects of pitavastatin and atorvastatin on high-density lipoprotein cholesterol levels and glucose metabolism in Japanese patients with elevated levels of low-density lipoprotein cholesterol and glucose intolerance. Clin Ther.

[B268] Coleman CI, Reinhart K, Kluger J, White CM (2008). The effect of statins on the development of new-onset type 2 diabetes: a meta-analysis of randomized controlled trials. Curr Med Res Opin.

[B269] Kettawan A, Takahashi T, Kongkachuichai R, Charoenkiatkul S, Kishi T, Okamoto T (2007). Protective effects of coenzyme q(10) on decreased oxidative stress resistance induced by simvastatin. J Clin Biochem Nutr.

[B270] Nadanaciva S, Dykens JA, Bernal A, Capaldi RA, Will Y (2007). Mitochondrial impairment by PPAR agonists and statins identified via immunocaptured OXPHOS complex activities and respiration. Toxicol Appl Pharmacol.

[B271] Sun W, Lee TS, Zhu M, Gu C, Wang Y, Zhu Y, Shyy JY (2006). Statins activate AMP-activated protein kinase in vitro and in vivo. Circulation.

[B272] Lai IR, Chang KJ, Tsai HW, Chen CF (2008). Pharmacological preconditioning with simvastatin protects liver from ischemia-reperfusion injury by heme oxygenase-1 induction. Transplantation.

[B273] Bao N, Minatoguchi S, Kobayashi H, Yasuda S, Kawamura I, Iwasa M, Yamaki T, Sumi S, Misao Y, Arai M, Nishigaki K, Takemura G, Fujiwara T, Fujiwara H (2007). Pravastatin reduces myocardial infarct size via increasing protein kinase C-dependent nitric oxide, decreasing oxyradicals and opening the mitochondrial adenosine triphosphate-sensitive potassium channels in rabbits. Circ J.

[B274] Gu W, Kehl F, Krolikowski JG, Pagel PS, Warltier DC, Kersten JR (2008). Simvastatin restores ischemic preconditioning in the presence of hyperglycemia through a nitric oxide-mediated mechanism. Anesthesiology.

[B275] Schafer C, Parlesak A, Eckoldt J, Bode C, Bode JC, Marz W, Winkler K (2007). Beyond HDL-cholesterol increase: phospholipid enrichment and shift from HDL3 to HDL2 in alcohol consumers. J Lipid Res.

[B276] Das SK, Vasudevan DM (2007). Alcohol-induced oxidative stress. Life Sci.

[B277] de la Monte SM, Yeon JE, Tong M, Longato L, Chaudhry R, Pang MY, Duan K, Wands JR (2008). Insulin resistance in experimental alcohol-induced liver disease. J Gastroenterol Hepatol.

[B278] Flanagan DE, Moore VM, Godsland IF, Cockington RA, Robinson JS, Phillips DI (2000). Alcohol consumption and insulin resistance in young adults. Eur J Clin Invest.

[B279] Goude D, Fagerberg B, Hulthe J (2002). Alcohol consumption, the metabolic syndrome and insulin resistance in 58-year-old clinically healthy men (AIR study). Clin Sci (Lond).

[B280] Bell RA, Mayer-Davis EJ, Martin MA, D'Agostino RB, Haffner SM (2000). Associations between alcohol consumption and insulin sensitivity and cardiovascular disease risk factors: the Insulin Resistance and Atherosclerosis Study. Diabetes Care.

[B281] Villegas R, Salim A, O'Halloran D, Perry IJ (2004). Alcohol intake and insulin resistance. A cross-sectional study. Nutr Metab Cardiovasc Dis.

[B282] Porte D, Baskin DG, Schwartz MW (2005). Insulin signaling in the central nervous system: a critical role in metabolic homeostasis and disease from C. elegans to humans. Diabetes.

[B283] Reagan LP (2007). Insulin signaling effects on memory and mood. Curr Opin Pharmacol.

[B284] Mastrocola R, Restivo F, Vercellinatto I, Danni O, Brignardello E, Aragno M, Boccuzzi G (2005). Oxidative and nitrosative stress in brain mitochondria of diabetic rats. J Endocrinol.

[B285] Urayama A, Banks WA (2008). Starvation and triglycerides reverse the obesity-induced impairment of insulin transport at the blood-brain barrier. Endocrinology.

[B286] Hou WK, Xian YX, Zhang L, Lai H, Hou XG, Xu YX, Yu T, Xu FY, Song J, Fu CL, Zhang WW, Chen L (2007). Influence of blood glucose on the expression of glucose trans-porter proteins 1 and 3 in the brain of diabetic rats. Chin Med J (Engl).

[B287] Kobayashi T, Matsumoto T, Kamata K (2005). The PI3-K/Akt pathway: roles related to alterations in vasomotor responses in diabetic models. J Smooth Muscle Res.

[B288] Taguchi A, White MF (2008). Insulin-like signaling, nutrient homeostasis, and life span. Annu Rev Physiol.

[B289] Nalam RL, Pletcher SD, Matzuk MM (2008). Appetite for reproduction: dietary restriction, aging and the mammalian gonad. J Biol.

[B290] Lane N (2008). Marine microbiology: origins of death. Nature.

[B291] Buttner S, Eisenberg T, Herker E, Carmona-Gutierrez D, Kroemer G, Madeo F (2006). Why yeast cells can undergo apoptosis: death in times of peace, love, and war. J Cell Biol.

[B292] Skulachev VP (1999). Phenoptosis: programmed death of an organism. Biochemistry (Mosc).

[B293] Webb C (2003). A complete classification of Darwinian extinction in ecological interactions. Am Nat.

[B294] Parvinen K (2005). Evolutionary suicide. Acta Biotheor.

[B295] al-Mahroos F, McKeigue PM (1998). High prevalence of diabetes in Bahrainis. Associations with ethnicity and raised plasma cholesterol. Diabetes Care.

[B296] al-Mahroos F, Al-Roomi K (1999). Overweight and obesity in the Arabian Peninsula: an overview. J R Soc Health.

[B297] Musaiger AO, Al-Mannai MA (2001). Weight, height, body mass index and prevalence of obesity among the adult population in Bahrain. Ann Hum Biol.

[B298] Narayan KM, Boyle JP, Thompson TJ, Gregg EW, Williamson DF (2007). Effect of BMI on lifetime risk for diabetes in the U.S. Diabetes Care.

[B299] Luchsinger JA, Reitz C, Patel B, Tang MX, Manly JJ, Mayeux R (2007). Relation of diabetes to mild cognitive impairment. Arch Neurol.

[B300] Young SE, Mainous AG, Carnemolla M (2006). Hyperinsulinemia and cognitive decline in a middle-aged cohort. Diabetes Care.

[B301] Esposito K, Giugliano F, Ciotola M, De SM, D'Armiento M, Giugliano D (2008). Obesity and sexual dysfunction, male and female. Int J Impot Res.

[B302] Bubliy OA, Loeschcke V (2005). Correlated responses to selection for stress resistance and longevity in a laboratory population of Drosophila melanogaster. J Evol Biol.

[B303] Rattan SI (2004). Aging, anti-aging, and hormesis. Mech Ageing Dev.

[B304] Byberg L, Melhus H, Gedeborg R, Sundstrom J, Ahlbom A, Zethelius B, Berglund LG, Wolk A, Michaelsson K (2009). Total mortality after changes in leisure time physical activity in 50 year old men: 35 year follow-up of population based cohort. BMJ.

[B305] Wijndaele K, Duvigneaud N, Matton L, Duquet W, Delecluse C, Thomis M, Beunen G, Lefevre J, Philippaerts RM (2009). Sedentary behaviour, physical activity and a continuous metabolic syndrome risk score in adults. Eur J Clin Nutr.

